# Benefits of Clinical Decision Support Systems for the Management of Noncommunicable Chronic Diseases: Targeted Literature Review

**DOI:** 10.2196/58036

**Published:** 2024-11-27

**Authors:** Klaudia Grechuta, Pedram Shokouh, Ahmad Alhussein, Dirk Müller-Wieland, Juliane Meyerhoff, Jeremy Gilbert, Sneha Purushotham, Catherine Rolland

**Affiliations:** 1 Boehringer Ingelheim International GmbH Ingelheim am Rhein Germany; 2 Adivus Medical Consultancy Mpv. Aarhus Denmark; 3 Department of Internal Medicine I University Hospital Aachen Aachen Germany; 4 Sunnybrook Health Sciences Centre University of Toronto Toronto, ON Canada; 5 PPD Australia Pty Ltd Sydney Australia; 6 Evidera, The Ark London United Kingdom

**Keywords:** clinical decision support system, digital health, chronic disease management, electronic health records, noncommunicable diseases, targeted literature review, mobile phone

## Abstract

**Background:**

Clinical decision support systems (CDSSs) are designed to assist in health care delivery by supporting medical practice with clinical knowledge, patient information, and other relevant types of health information. CDSSs are integral parts of health care technologies assisting in disease management, including diagnosis, treatment, and monitoring. While electronic medical records (EMRs) serve as data repositories, CDSSs are used to assist clinicians in providing personalized, context-specific recommendations derived by comparing individual patient data to evidence-based guidelines.

**Objective:**

This targeted literature review (TLR) aimed to identify characteristics and features of both stand-alone and EMR-integrated CDSSs that influence their outcomes and benefits based on published scientific literature.

**Methods:**

A TLR was conducted using the Embase, MEDLINE, and Cochrane databases to identify data on CDSSs published in a 10-year frame (2012-2022). Studies on computerized, guideline-based CDSSs used by health care practitioners with a focus on chronic disease areas and reporting outcomes for CDSS utilization were eligible for inclusion.

**Results:**

A total of 49 publications were included in the TLR. Studies predominantly reported on EMR-integrated CDSSs (ie, connected to an EMR database; n=32, 65%). The implementation of CDSSs varied globally, with substantial utilization in the United States and within the domain of cardio-renal-metabolic diseases. CDSSs were found to positively impact “quality assurance” (n=35, 69%) and provide “clinical benefits” (n=20, 41%), compared to usual care. Among CDSS features, treatment guidance and flagging were consistently reported as the most frequent elements for enhancing health care, followed by risk level estimation, diagnosis, education, and data export. The effectiveness of a CDSS was evaluated most frequently in primary care settings (n=34, 69%) across cardio-renal-metabolic disease areas (n=32, 65%), especially in diabetes (n=13, 26%). Studies reported CDSSs to be commonly used by a mixed group (n=27, 55%) of users including physicians, specialists, nurses or nurse practitioners, and allied health care professionals.

**Conclusions:**

Overall, both EMR-integrated and stand-alone CDSSs showed positive results, suggesting their benefits to health care providers and potential for successful adoption. Flagging and treatment recommendation features were commonly used in CDSSs to improve patient care; other features such as risk level estimation, diagnosis, education, and data export were tailored to specific requirements and collectively contributed to the effectiveness of health care delivery. While this TLR demonstrated that both stand-alone and EMR-integrated CDSSs were successful in achieving clinical outcomes, the heterogeneity of included studies reflects the evolving nature of this research area, underscoring the need for further longitudinal studies to elucidate aspects that may impact their adoption in real-world scenarios.

## Introduction

Clinical decision support systems (CDSSs) are designed to assist health care professionals (HCPs) in making evidence-based decisions using patient information, clinical knowledge, and other health care data [1]. CDSSs are typically developed as computer-based tools or software applications and can function independently (termed stand-alone) or be connected to an electronic medical record (EMR) database (termed EMR-integrated systems). According to regulations for software as medical devices in Canada and the United States and a medical device regulation in the European Union [2,3], a CDSS can be classified as a medical device or can be exempt from regulations as a nonmedical device, depending on its specific functions and intended purpose; however, the specific criteria and regulations may differ across countries or regions.

CDSSs are used to provide support across a spectrum of health care activities, including early disease detection, diagnosis, data interpretation, treatment planning, patient care, monitoring, and prognosis [4]. These functions can be enabled via specific CDSS features, such as the ability to estimate treatment efficacy or level of risk, recommend a treatment regimen or a referral, predict drug-drug interactions and dosing alerts, flag potential aspects for further monitoring, and facilitate shared decision-making [5,6]. CDSSs can be knowledge based, using predefined clinical decision rules (such as reinforcement-based methods), or non–knowledge based, relying on data-driven approaches like artificial intelligence, machine learning, or statistical pattern recognition, instead of being programmed to follow expert medical knowledge [7]. CDSSs can integrate data from multiple sources such as clinical guidelines, peer-reviewed literature, historical patient data, and EMRs [4]. In integrated CDSSs, input data (ie, relevant patient information) are acquired from an EMR database, such as Epic [8], Allscripts [9], Aifred Health [10], and Eclinicalworks [11], which serve as primary platforms for clinicians to check patient laboratory results, clinical documentation, and reports [4]. In contrast, stand-alone systems require manual data entry such as Epocrates [12], a CDSS that provides accurate, reliable, and actionable clinical tools to support clinicians in making point-of-care decisions [4].

CDSSs aim to improve health care delivery by enhancing medical decisions and tailoring patient care with targeted clinical knowledge and multidimensional medically relevant health indicators. These indicators can span across several dimensions of patient health and include but are not restricted to vital signs (eg, blood pressure and heart rate), laboratory values (eg, blood glucose levels and cholesterol levels), diagnostic test results (eg, electrocardiogram and magnetic resonance imaging), disease-specific biomarkers (eg, tumor markers and hemoglobin A_1c_ [HbA_1c_]), patient-specific data (eg, allergies and medications), and predictive models (eg, Framingham risk score). Since CDSSs first originated in the 1970s, a manifold of systems has been developed and implemented using different modalities (eg, PC and tablet) as EMR-integrated or stand-alone systems for the management of numerous conditions at different stages, with a vast scope of functions (flagging, drug control, shared decision-making, etc), users (eg, HCPs, patients, and nurses), and settings (eg, ambulance, surgery room, and general practitioner’s office) to address unmet needs of a given region or country [13]. In the context of ongoing digital transformation in the health care sector, the potential of CDSSs for improving the quality and efficacy of care has been increasing. Indeed, CDSSs have been associated with a positive impact on the quality of clinical practice and patient adherence in the management of chronic diseases [4,6,14]. It is noteworthy that while some CDSS significantly improved clinical practice, some yielded limited results, and others failed to meet expectations [15-17]. To date, a body of research sheds light on the reasons behind the lack of success or adoption of CDSSs from both theoretical and practical standpoints [6,16]; however, while previous research addresses clinical practice in general terms for both computer-based and manual systems, this review aims to complement previous work by focusing on the studies that address electronic and guideline-based CDSSs for the management of chronic, noncommunicable diseases in a primary care setting. Specifically, we aim to identify features and characteristics that impact the efficacy of CDSSs based on the outcome measures, consequently contributing to several value areas. We sought to gain a deeper understanding of the various value areas where improvements could be made through these systems, as well as the features that contributed to positive outcomes in the studies evaluating the benefits of CDSSs for HCPs.

## Methods

### Overview

This targeted literature review (TLR) was conducted to identify features of stand-alone and EMR-integrated CDSSs that positively impacted multiple value areas (Multimedia Appendix 1) and overall success (as measured by respective clinical outcomes) as reported in peer-reviewed literature. The TLR was conducted using multiple sources, including the Embase, MEDLINE, and Cochrane databases, and searches were limited to literature published from January 2012 to October 2022. The searches were performed using a combination of free-text search terms and controlled vocabulary terms specific to each database (Tables S1-S4 in Multimedia Appendix 2). Only studies published in the English language were eligible for inclusion. The TLR searches were conducted with no geographical restriction. All conferences indexed in Embase were searched to identify relevant abstracts published within a 2-year timeframe from January 2020 to October 2022. The search strategies are detailed in Tables S1-S4 in Multimedia Appendix 2.

The eligibility criteria were developed based on population or scope of interest, intervention, comparators, outcomes, and study design (Table 1). The scope of interest was all health care providers. The TLR included studies on computerized, guideline-based CDSSs with a focus on chronic disease areas. Clinical trials, as well as observational studies (ie, cohort, case-control, cross-sectional, and case series) conducted using CDSSs with HCPs across chronic noncommunicable disease areas were included in the TLR. Narrative reviews, conference abstracts published before 2020, and any CDSSs that were not computerized (based on software application) or not based on guidelines were excluded. Studies reporting evidence for CDSSs from the patient benefit perspective or studies in patient-oriented, self-management tools [18] were out of the scope of this review.

**Table 1:** Study selection criteria.

	Inclusion criteria	Exclusion criteria
Population or scope of interest	Guideline-based CDSS^a^ for care and treatment management Disease area: chronic, metabolic, or noncommunicable etiology Use group (including but not restricted to PCPs^b^, nurse practitioners, nurses, specialists, pharmacists, medical students, educators, patients, and caregivers)	Decision support systems that are not computerized or guideline based Disease areas (duration of disease [acute], natural course of disease [curable], and non–disease-specific CDSSs) User group is out of scope (eg, patients)
Intervention or approach of interest	CDSS	—^c^
Comparator	Any or none	—
Outcomes or variables of interest	Features of CDSSs that impact the successful adoption of these systems (including but not restricted to system type: stand-alone or incorporated in an EMR^d^) Value areas (clinical benefits, patient safety and risk, workflow improvements, educational aspects, user satisfaction, quality assurance, guideline adherence, and patient behavioral change or self-management) Investigational or marketed device? Main function of the CDSS in disease management (treatment management only or treatment management + combined diagnostic) Study setting (primary health care setting, hospital outpatient setting, community health care setting, or rehabilitation clinic)	Intended purpose of the CDSS is out of scope (blood transfusion, antibiotics treatment, treatment of attacks [including stroke, management of infections, fractures or pain, and support for organ transplants], or systems used in diagnostic purposes exclusively [eg, screening or testing only]) Diseases are out of scope (eg, dermatology and rheumatology) Study setting (inpatient, ICU^e^, or emergency department)
Study design (full-text publications only)	Observation studies (ie, cohort, case-control, cross-sectional, or case series) Randomized and nonrandomized studies Database studies Modelling studies Economic evaluations SLRs^f^ (for reference chasing only)	Qualitative studies (eg, interviews) Conference abstracts SLRs Narrative reviews Methods or protocol papers
Geography	Global, with a specific focus on Canada, the United States, and Germany	No restriction
Timeframe	10 years (from January 212 to October 2022)	Studies published before January 2012

^a^CDSS: clinical decision support system.

^b^PCP: primary care provider.

^c^Not applicable.

^d^EMR: electronic medical record.

^e^ICU: intensive care unit.

^f^SLR: systematic literature review.

### Study Selection

Following the searches, all identified studies were screened for inclusion by a single reviewer and verified by other CDSS domain experts (KG and PS) by reviewing the title and abstract, based on predefined study eligibility criteria (Table 1). Selected studies then underwent full-text screening by a single reviewer, followed by CDSS domain expert (KG and PS) verification. The TLR used a comprehensive approach by tagging information at the full-text screening stage to help narrow the scope and prioritize key evidence needed to answer the research question. Tagging captured information on categorical variables such as disease area, CDSS features, value area or areas (as shown in Table 1), methods used to implement CDSS, target user groups, product category (investigational or marketed CDSS), function of CDSS in disease management, and environment of use. All categories of tagging are detailed in Table S5 in Multimedia Appendix 2. The tagging information was quality-checked for accuracy by 2 other reviewers (CR and KG). Any disagreements between reviewers about screening decisions were resolved by CDSS researchers (KG and PS). Studies that met all inclusion criteria and none of the exclusion criteria were eligible for inclusion in the review.

### Data Extraction

A data extraction form was designed and developed in Excel (Microsoft Corp), which included key variables such as study objectives, study design, CDSS tool details, disease area, outcome measurement, CDSS value areas, CDSS features, methods of CDSS integration, and study conclusions, among others. An exhaustive list of all data of extracted variables is provided in Table S6 in Multimedia Appendix 2. The design of the data extraction form, selection of variables for extraction, classification of value areas (Multimedia Appendix 1), classification of features (Multimedia Appendix 3), and classification of disease areas (Multimedia Appendix 4) were iterative processes supported by literature classification frameworks within existing CDSS literature and inputs from CDSS domain experts (PS, KG, and AA). Furthermore, in alignment with available literature [6,16], the selected CDSS studies were heterogeneous in terms of architecture, modality, setting, input and output data, functionalities, type of integration, and intended user group, which provided additional insights into identifying the variables of interest. To compare the included CDSSs in an unbiased manner, we screened all studies for common differentiators, which, in addition to the established criteria, further informed the design of the data extraction framework. CDSSs evaluated in the reviewed studies were classified as successful or unsuccessful. Success was defined as having achieved the study outcomes and met the study objectives. To evaluate the performance of CDSSs as reported in the identified studies, we extracted information pertaining to their “objectives” and “success” for each study. We defined 3 criteria of success based on whether a given study met primary and secondary objectives (ie, successful study), did not meet any objectives (ie, unsuccessful study), or met some of the goals (ie, partially successful study). An example of meeting set objectives includes rejecting the null hypothesis in the case of randomized controlled trials (RCTs) by showing improved cardiovascular risk associated with the utilization of a CDSS [19] or acquiring desirable scores on satisfaction scales or positive qualitative feedback from HCPs in the case of an observational study [20]. While not fully explored in this review, another measure of the success of a CDSS is adoption (or uptake), as it indicates whether the system is fulfilling its intended purpose in practical, everyday health care settings. Data were extracted into the form by one investigator (SP) and further verified by a second investigator (CR). The completed data extraction form was validated for accuracy and completeness by two additional domain experts (PS and KG). The results of the validated data extraction were analyzed using a narrative approach to synthesize the findings of the review.

## Results

### Literature Search Results

**Figure 1:**
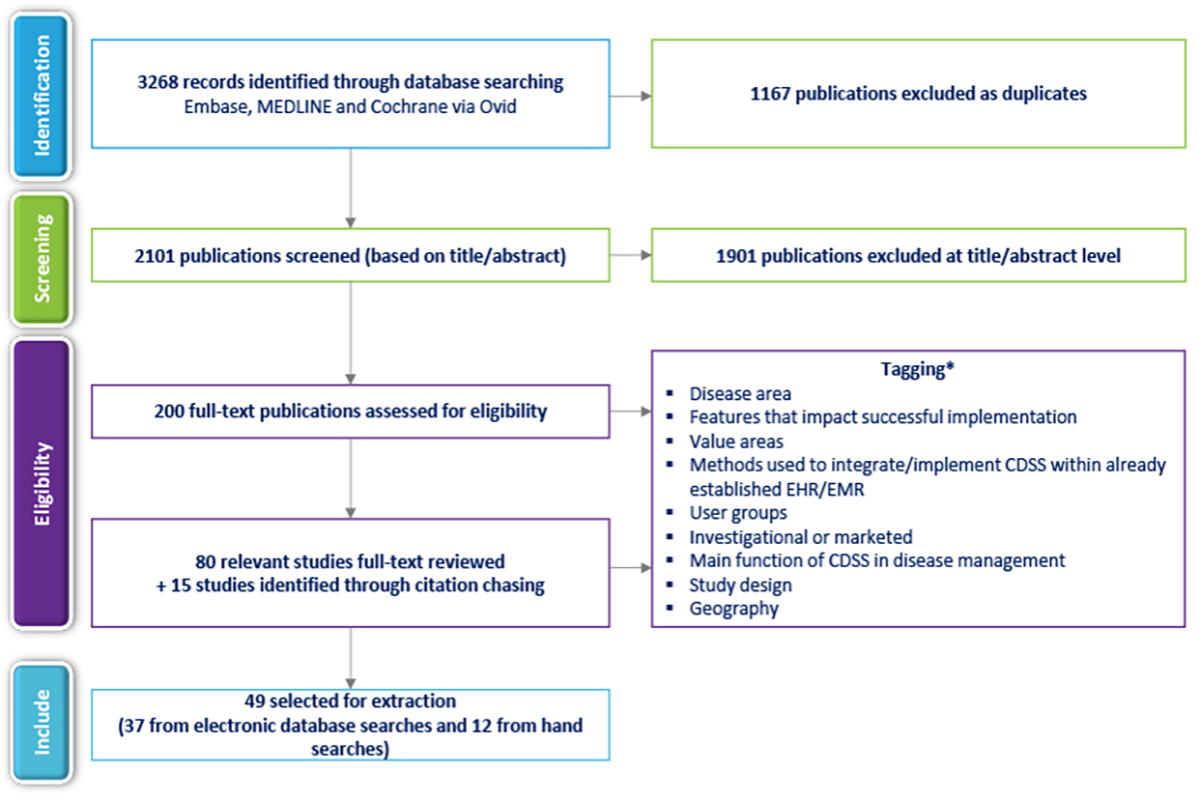
TLR flowchart. CDSS: clinical decision support system; EHR: electronic health record; EMR: electronic medical record; TLR: targeted literature review. *Examples of categories are provided in Table S5 in Multimedia Appendix 2.

The electronic database searches yielded 3268 records. After removing 1167 duplicates, 2101 unique records were screened by reviewing titles and abstracts. Of these, 80 publications met the eligibility criteria for full-text screening (Figure 1). Figure 1 summarizes the complete screening process in a TLR flow diagram. Following a full-text review, 49 unique records met the study eligibility criteria and were included in the review (Multimedia Appendix 5 [19-67]). Of these, 37 full-text publications were identified from electronic searches, and 12 were identified through manual searches of reference lists of journal studies.

### Study Characteristics

The TLR included RCTs (n=24, 49%), nonrandomized studies (n=2, 4%), and observational studies (n=23, 47%). The studies were performed in various environments of use, and primary care settings (n=34, 69%) were represented most frequently, followed by specialty clinics (n=7, 14%), and hospital outpatient settings (n=7, 14%); one study did not report study settings. The disease areas of CDSSs varied across studies, and cardiorenal diseases were reported most frequently (n=32, 65%), followed by diabetes (n=13, 56%) and atrial fibrillation (n=5, 10%).

### Stand-alone Versus EMR-Integrated CDSS

Of the 49 included studies, 32 (65%) studies evaluated CDSSs integrated into EMRs, while 17 (35%) studies were on stand-alone CDSSs (ie, operating independently).

#### Geographic Distribution

The included studies were conducted across diverse geographical locations, spanning a total of 15 countries (Figure S1 in Multimedia Appendix 2). Of the 17 studies on stand-alone CDSSs (17/49, 35% of all studies), 6 (35% of the 17 stand-alone studies) were conducted in the United States, and the others originated from Austria (1/17, 6%), Brazil (1/17, 6%), Canada (2/17, 12%), China (1/17, 6%), Iceland (2/17, 12%), India (1/17, 6%), the Netherlands (1/17), and the United Kingdom (1/17, 6%; Figure S2 in Multimedia Appendix 2). One stand-alone study was a multicenter study conducted in Austria, Germany, Italy, and the United Kingdom. Of the 32 EMR-integrated CDSS studies (32/49, 65% of all studies), 15 (46% of the 32 EMR-integrated studies) were conducted in the United States, followed by Canada (3/32, 9%), the Netherlands (2/32, 6%), and China (2/32, 6%); 1 (3%) study originated from each, Australia, Sweden, Brazil, Belgium, India, Spain, Sri Lanka, United Kingdom, and Singapore (Figure S2 in Multimedia Appendix 2), and 1 (3%) EMR-integrated CDSS study was a multicenter study conducted in India and Pakistan.

#### Disease Areas

Stand-alone CDSSs, as well as integrated CDSSs, were widely used in the management of type 2 diabetes (stand-alone: 5/17, 29%; integrated: 8/32, 25%). Additionally, both types of CDSSs were used in managing cardiovascular diseases (CVD), such as atrial fibrillation (stand-alone: 1/17, 6%; integrated: 4/32, 13%). Stand-alone CDSSs were used to manage CVDs such as heart failure (1/17, 6%) and cardiac rehabilitation (1/17, 6%; Figure S3 in Multimedia Appendix 2), while integrated CDSSs were used in managing CVDs such as atherosclerotic CVD (2/32, 6%; Figure S4 in Multimedia Appendix 2). Only a limited number of CDSSs were used in cancer management (stand-alone: 1/17, 5.9%; integrated: 4/32, 13%). Fewer CDSSs focused on disease areas including mental health (stand-alone: 2/17, 12%; integrated: 3/32, 9%), respiratory (stand-alone: 1/17, 6%; integrated: 1/32, 3%), musculoskeletal (stand-alone: 2/17, 12%; integrated: 0/32, 0%), and neurogenic conditions (stand-alone: 0/17, 0%; integrated: 2/32, 6%). One CDSS supported multiple chronic conditions such as hypertension, diabetes mellitus, and depression (stand-alone: 0/17, 0%; integrated: 1/32, 3%).

#### Environment of Use

Both CDSS types were extensively used in primary care settings in 69% (34/49) of studies (stand-alone: 11/17, 65%; integrated: 23/32, 74%), followed by specialty clinics in 14% (7/49) of studies (stand-alone: 4/17, 23%; integrated: 3/32, 10%), and hospital outpatient settings in 14% (7/49) of studies (stand-alone: 2/17, 12%; integrated: 5/32, 16%). One study did not report the study settings.

#### Modality of Use and Data Sources

Both types of CDSS used various modalities, ranging from desktop applications on computers to smartphones and tablets. Several integrated CDSSs, as well as stand-alone CDSSs, were only accessible via desktop computer platforms (stand-alone: 11/17, 65%; integrated: 29/32, 90%). Stand-alone CDSSs were delivered as smartphone apps across 23% (4/17) of studies compared to 6% (2/32) of studies with integrated CDSSs. One stand-alone CDSS was also deployed on a tablet (6%); however, no tablet-based EMR-integrated CDSS was reported. Across studies, combinations of manual, automated, and mixed input data types were reported. The majority of EMR-integrated CDSSs were interpreted as being fully automated (25/32, 78%), and in some cases, they combined both manual and automated data entry (6/32, 19%); one study reported on a manual CDSS that included a form of a questionnaire to be filled by patients, whereby the data were processed to the CDSS by HCPs to generate recommendations. In a large proportion of stand-alone CDSSs (9/17, 53%), data were provided by the HCPs who manually entered relevant information into the system to generate recommendations. Finally, 18% (3/17) of CDSSs were automated systems continuously monitoring patient data to provide real-time decision support, and some were a mix of manual and automated systems (4/17, 23%; one study did not provide this information).

Information on whether the CDSS was still in the research-and-development phase or fully developed and commercially available was lacking in 78% (38/49) of the studies (stand-alone: 17/17, 100%; integrated: 21/32, 66%). Overall, only 12% (6/49) of CDSSs, especially those integrated with EMRs (stand-alone: 0/17, 0%; integrated: 6/32, 19%), were understood to be commercially available or ready to be used in health care settings.

#### User Groups

CDSSs were found to target diverse user groups based on a broad spectrum of roles and accountabilities across the health care ecosystem. The key user groups identified through the TLR were combinations of physicians, nurses or nurse practitioners, medical assistants, and care coordinators in 55% (27/49) of studies. Primary care physicians were the sole users of CDSSs in 33% (16/49) of CDSS studies. Meanwhile, other studies indicated exclusive use by specialists, including cardiologists (1/49, 2%), nephrologists (2/49, 4%), and oncologists (2/49, 4%). This pattern of mixed CDSS users was reflected across stand-alone and integrated types of CDSSs (mixed users: stand-alone CDSSs 11/17, 65%; EMR-integrated CDSSs 16/32, 50%). Among mixed user groups, a group of physicians and nurses (stand-alone: 5/17, 46%; EMR-integrated: 11/32, 65%) was most frequently reported across both stand-alone and EMR-integrated CDSS studies. Primary care physicians were the exclusive user group in 29% (5/17) of stand-alone and 34% (11/32) of EMR-integrated studies.

#### Value Areas

CDSSs provide value across the diverse domains of health care systems, including quality assurance, clinical benefits, guideline adherence, user satisfaction, patients’ safety or risk management, workflow improvement, patient behavior, educational aspects, and financial aspects (Table 2). Value areas in the TLR were linked to the outcome measures reported in the studies. The studies evaluated the effectiveness of CDSSs by assessing their impact on a range of value areas. These value areas collectively demonstrated the broad range of CDSS benefits across all studies identified in the TLR. The value areas are described in Multimedia Appendix 1. Quality assurance stood out as a prominent value area reported across 69% (35/49) of studies, followed by clinical benefits (20/49, 41%) and user satisfaction (20/49, 41%). The primary value areas were similar in stand-alone and integrated CDSS, which most frequently were reported to contribute to improving quality assurance, clinical benefits, user satisfaction, and guideline adherence.

**Table 2:** Value areas identified across stand-alone and integrated CDSSs^a^.

	EMR^b^-integrated studies (n=32), n (%)^c^	Stand-alone studies (n=17), n (%)
Quality assurance	23 (72)	12 (71)
Clinical benefits	15 (44)	5 (29)
User satisfaction	14 (44)	6 (35)
Guideline adherence	9 (28)	5 (29)
Workflow improvements	7 (22)	3 (18)
Educational aspects	4 (13)	1 (6)
Patient behavior or self-management	3 (9)	2 (12)
Patient safety and risk	2 (6)	4 (24)
Financial aspects	2 (6)	0 (0)

^a^CDSS: clinical decision support system.

^b^EMR: electronic medical record.

^c^Percentages indicate the proportion of studies reporting on the respective value area. The percentages do not add up to 100%, as one study may report on more than one value area.

### CDSS Key Features

CDSSs offer a variety of features, each contributing to key value areas (Multimedia Appendix 1). These features include the ability to flag potential issues for screening and follow-up; ensure safety by monitoring for incorrect indications; and provide support for treatment management, including estimating efficacy, recommending dosages, and suggesting pharmacotherapy options. The features of CDSSs may also facilitate shared decision-making, allow for result exporting, offer screening recommendations, support monitoring, provide referral recommendations, estimate risk levels, aid in diagnosis, contribute to patient education, and enable audits to identify deviations from recommended clinical pathways. Features pertaining to treatment recommendation and flagging were interpreted to be most used across all value areas identified in the TLR. Across stand-alone CDSSs, the most used feature was treatment recommendation by way of assisting HCPs in selecting appropriate pharmacological options; however, across integrated CDSSs, the features of flagging related to noncompliance to guideline-directed therapy [21], treatment eligibility [22], and screening with follow-up [23] were the most beneficial in enhancing all value areas.

### Key Features of Successful and Unsuccessful CDSSs

#### Successful CDSSs

Over two-thirds (34/49, 69%) of all CDSSs identified in the TLR achieved the study outcomes and met the study objective, thus they were categorized as successful in the TLR. Approximately 8% (4/49) of CDSS studies were found to have achieved some (≥1) study outcomes and fallen short in other areas and were considered partially successful. The majority of stand-alone CDSSs (13/17, 76%), as well as EMR-integrated CDSSs (21/32, 66%), achieved their objectives. For the stand-alone CDSSs, 12% (2/17) were deemed unsuccessful and another 12% (2/17) were classified as partially successful. These were reported as 16% (5/32) and 19% (6/32), respectively, for the EMR-integrated CDSS.

**Figure 2:**
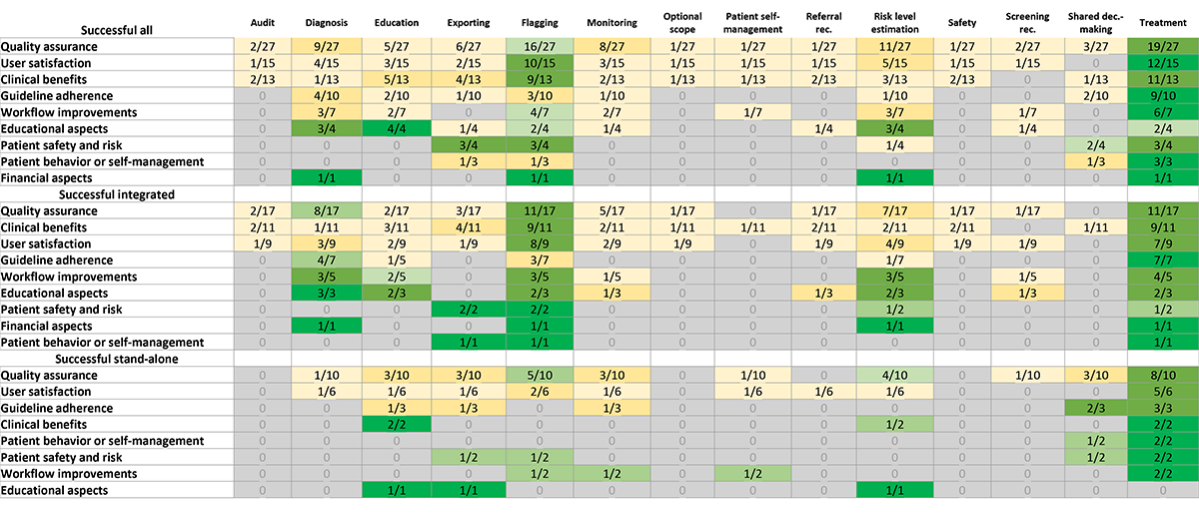
Features of CDSSs for all successful studies. CDSS: clinical decision support system; dec.: decision; rec.: recommendation.

Of the 34 successful CDSSs, positive impacts on value areas were reported across all studies, and quality assurance was reported most frequently (n=24, 71%), followed by user satisfaction (n=13, 38%), and clinical benefit (n=15, 44%). Other value areas such as guideline adherence (6/34, 18%), workflow improvement (6/34, 18%), patient safety and risk (4/34, 12%), educational aspects (2/34, 6%), patient’s behavioral change (3/34, 9%), and financial aspects (2/34, 6%) were less frequently reported across successful CDSSs. Treatment recommendations and flagging were the features that emerged as the most frequent for successful studies across all the value areas (Figure S1 in Multimedia Appendix 6). Flagging typically involved the CDSS highlighting specific information or events to the attention of the HCPs. Flagging across included CDSS studies encompassed various types, such as flagging to notify of best practice advice, clinical guideline adherence, eligibility for preventative screening and follow-up, glycemic target deviation in diabetes, safety monitoring, out-of-range patient data, and flagging for incorrect indications. The flagging feature played a more important role in patient safety and risk than in any other value area across all successful CDSSs (Figure S1 in Multimedia Appendix 6). Similarly, treatment recommendation was also a frequently used feature in ensuring guideline adherence across all successful CDSS studies included in the TLR (Figure S1 in Multimedia Appendix 6). Other frequently reported features included risk level estimation, diagnosis, education, data export, and monitoring (Figure S1 in Multimedia Appendix 6).For successful stand-alone CDSSs, the most frequent feature was treatment recommendation, which was crucial across all value areas except educational aspects (Figure S1 in Multimedia Appendix 6). This feature played a central role in assisting health care systems and impacting multiple value areas such as patient behavior or self-management, guideline adherence, quality assurance, and user satisfaction. CDSS features such as referral recommendations and screening recommendations were less commonly used than other features (Figure 2).

Across studies on successful EMR-integrated CDSSs, the flagging feature was particularly notable as the most reported feature that contributed to multiple value areas such as quality assurance, clinical benefits, user satisfaction, and patient safety and risk management (Figure S2 in Multimedia Appendix 6). Additionally, alongside flagging, studies on successful EMR-integrated CDSSs also reported treatment recommendations, especially to ensure guideline adherence (Figure S3 in Multimedia Appendix 6).

#### Unsuccessful CDSSs

Unsuccessful CDSS studies were reported to have achieved neutral or negative study outcomes across measured value areas. Only a small number of CDSSs (7/49, 14%) failed to meet the study objectives, including 16% (5/32) of integrated CDSSs and 12% (2/17) of stand-alone CDSSs. Across unsuccessful CDSS studies, flagging and treatment recommendations were also the features most frequently reported across value areas (Figure S5 in Multimedia Appendix 2). The flagging feature was present across value areas comprising patient safety and risk management, quality assurance, and guideline adherence. The treatment recommendation feature was reported in relation to guideline adherence, quality assurance, patient safety and risk management, and patient behavior. Table 2 summarizes the study characteristics of 49 CDSS studies.

## Discussion

### Key Findings

This TLR aimed to provide a comprehensive assessment of CDSSs reported in peer-reviewed literature. The specific objective was to identify features of CDSSs that positively impact medically relevant value areas and the overall study success as measured by corresponding clinical outcomes.

According to our findings, in most studies, the application of a CDSS was associated with added clinical value for HCPs. A positive impact of CDSSs was reported for quality assurance in patient care, in particular by improving adherence to clinical guidelines, as well as better diagnostic support, monitoring practices, and additional opportunities for preventative screening and treatment. Using CDSSs was also associated with important clinical benefits, including improved accuracy in diagnoses, clinical decision-making, treatment selection, and thereby, enhanced patient outcomes.

Studies examined in this TLR offered valuable insights into the functions of an effective CDSS depending on its intended purpose. Besides features of successful CDSSs, the studies frequently reported that irrelevant and interruptive alerts led to so-called “alert fatigue” and low use rates, consequently leading to the failure of CDSSs [21,24,25]. These insights underscore the importance of prompting clinician action, integrating CDSSs seamlessly into workflows, and mitigating alert fatigue, all of which are crucial factors for the successful adoption of a CDSS [21,24,25]. A study by Ballard et al [68] found that incentivizing HCPs to use the tool through financial bonuses and other promotional incentives resulted in an increase in CDSS use rates. While the design of a CDSS is a fundamental criterion, other factors such as the organizational and technological infrastructure of the primary care system can also markedly influence the success of a CDSS [24].

The review found that the type of CDSS was not a crucial factor for its success, as both integrated and stand-alone CDSSs contributed to improved outcomes and were identified as beneficial for use in health care. While EMR-integrated CDSSs may have the advantage of ongoing access to patient data in real-time, stand-alone CDSSs offer the flexibility of operating independently from other systems, portability, and faster deployment (ie, the process of making the CDSS available and operational across the health care system) [69-71]. Therefore, the choice between a stand-alone or integrated CDSS should be guided by the needs and preferences of HCPs, intended use and application settings, regulatory considerations, and goals for improving patient care.

### Successful CDSSs

Flagging was a feature in 59% of the studies reporting successful CDSSs and emerged as a pivotal feature, especially in the context of patients’ safety and risk management (Figure S1 in Multimedia Appendix 6). Flagging features were used in a variety of scenarios including notifications of best practice advice or clinical guideline adherence, eligibility for preventative screening, glycemic target deviation in diabetes, screening and follow-up, safety monitoring, out-of-range patient data, and incorrect indications. Other value areas such as clinical benefits, user satisfaction, and quality assurance were also frequently enhanced by the flagging feature. In addition to flagging, treatment recommendations (including recommendations for pharmacotherapy, dosage optimization, and nonpharmacological therapy) also played a notable role across multiple value areas, especially regarding guideline adherence. Although less frequently reported, successful CDSSs offered a range of other features beyond flagging and treatment recommendations, including risk level estimation, diagnosis suggestions, and the ability to export information. The popularity of these features may be explained by the specific needs of health systems, health care providers, or disease areas. Therefore, the effectiveness of a CDSS may often rely on a combination of features that collectively contribute to improved patient care, safety, and clinical status. Developers of CDSSs should consider the specific needs and priorities of HCPs in terms of target value areas when designing and implementing a CDSS to ensure that it addresses the unmet need and is effectively and seamlessly integrated within the HCPs’ workflow.

### Unsuccessful CDSS

Only 7 (14%) studies identified in this review reported CDSSs to be unsuccessful, that is, failing to fulfill study outcomes, compared with conventional approaches to disease management. Similar to successful studies, unsuccessful studies commonly reported the utilization of flagging and treatment recommendation features. Flagging was most often reported to address patient safety and risk management, while treatment recommendation was reported to address guideline adherence in unsuccessful studies. There were no recognizable aspects among unsuccessful CDSS studies that may have contributed to their failure. While unsuccessful CDSSs did not achieve the study objectives, it is important to note that the efficacy of a CDSS may vary depending on various factors, which this review found very little data on (only 7 CDSS studies failed). The included publications did not provide sufficient granularity to fully understand the reasons for which the CDSSs were not successful; however, the authors suggested that the failure of a CDSS was mostly attributed to ineffective design, low use rate, organizational challenges, and issues with CDSS alerts. The authors also highlighted the importance of rigorous testing and undertaking quality improvement initiatives to ensure the effectiveness of a CDSS.

### Importance of the Findings

The findings of our review suggest that CDSSs can improve screening for CVD risk factors, such as diabetes, as well as quality assurance and user satisfaction, resulting in clinical benefits for the management of cardio-renal-metabolic diseases. This is of particular interest to the development of novel CDSSs in this disease area such as Exandra, a tool supporting Canadian HCPs in the management of type 2 diabetes by offering individualized, patient-specific recommendations based on the Diabetes Canada Clinical Practice Guidelines [72] (Exandra as a product has been decommissioned; however, the recommendation engine that supported Exandra is being used under different names in Canada and other countries).

The success of a CDSS may not always result in its adoption across studies. Adoption measures for the uptake of a CDSS in health care settings were largely underreported and inadequately described across successful CDSS studies. Only two studies reported some plans in place for the successful adoption of CDSS in health care settings. A single-arm, longitudinal, observational study conducted in Brazil on HealthRise, a CDSS designed for diabetes and hypertension management in primary care, reported plans to expand the CDSS to primary care units in other towns, include more diseases, and implement it in various settings. Efforts were also underway to address the challenge of integrating the CDSS with the Brazilian public health system’s Electronica-Sistema Unico de Saude. Further, a retrospective study conducted in the United States included a CDSS prototype built on the drools platform that can interpret Papanicolaou test (Pap) reports and provide cervical cancer screening and management guidelines. The authors reported that after physician validation, the developed CDSS would be deployed in the Mayo Clinic’s outpatient departments with user feedback collected on its performance. The CDSS will integrate with an EMR interface listing all preventive care reminders. An impact analysis was also planned to compare screening and referral rates before and after deployment. It should be noted that the assessment of the successful adoption of a CDSS, as well as the identification of key attributing factors, may have been compromised by the heterogeneity of outcomes evaluated across studies and by the study design (ie, RCTs, whereby participants were incentivized to use a given system). RCTs in the TLR focused predominantly on the efficacy of CDSSs in improving quality assurance and other value areas as the primary outcome, with less emphasis on reporting planned adoption measures for their CDSSs. In contrast, a few observational studies noted challenges with CDSS adoption related to scaling, portability, generalizability of results, limited transferability to different settings, and resource constraints. Among the CDSS studies included in the review, only two observational studies confirmed adoption to health care settings. The primary reasons for the deployment of CDSSs included a high level of CDSS accuracy, user satisfaction, and the potential to improve guideline adherence. Deployment included essential measures to optimize CDSS performance such as user feedback, impact analysis, and intervention monitoring and refinement. Therefore, real-world evidence from marketed or integrated CDSSs, as well as insights from longitudinal observational studies encompassing diverse perspectives, may shed further light on features that contribute to long-term adoption. It is important to note that successful CDSS adoption depends on a combination of factors, including alignment with objectives of HCPs, workflow integration, clinical relevance, usability, customization, training and support, interoperability, technical expertise, resource constraints, financial viability, and multi-level engagement, as well as governmental regulations and barriers [5,73-75]. Active engagement and collaboration by the end users, and in particular, HCPs, to address challenges are vital to ensure that a CDSS meets its objectives [76].

### Comparison With Previous Reviews

Our findings show that CDSSs improved quality assurance and user satisfaction, and they provided clinical benefits in cardio-renal-metabolic conditions, though heterogeneity in population characteristics, interventions, and outcomes limits the generalizability of these findings. A recent systematic review on the impact of CDSS on clinical and patient-reported outcomes in patients with chronic disease found inconclusive evidence, primarily attributed to the heterogeneity and methodological biases of included studies. Similarly, a previous review on the effect of CDSS on cardiovascular risk factors found no definitive clinical benefits but highlighted the clinical relevance of CDSS in enhancing shared decision-making [77]. Both reviews emphasize the heterogeneity of studies and methodological differences that reflect the immaturity and the evolving nature of research in the field [77,78].

Previous reviews on the impact of CDSSs in multiple disease areas including CVD and diabetes report a positive effect on physician performance, such as adherence to clinical guidelines and prescription of drugs, whereas they had no impact on other outcomes [4,79,80]. Evidence demonstrating the positive impact of CDSS on prescribing treatments, facilitating preventative care services, and ordering laboratory tests across diverse venues was reported in another review [81]. This study builds on previous findings and has identified key features, such as flagging and treatment recommendations, which are associated with improved patient outcomes. This is in agreement with a previous review assessing features of CDSSs, which identified that features that provided support as part of clinician workflow and those that provided actionable recommendations were strongly associated with CDSS’s ability to improve clinical practice [6].

### Study Limitations

The TLR followed robust, protocol-driven methods, ensuring a structured and rigorous review process. However, it should be acknowledged that there are no standardized gold standard methods for conducting a TLR; reviews can range from very rapid and targeted searching to comprehensive mimicking a systematic review (but using only one reviewer), which was the case with this TLR. While single screening may increase the potential risk of bias, this was effectively mitigated by leveraging the expertise of subject matter experts to validate the decisions. In addition, it is important to note limitations within the evidence itself. CDSS studies exhibited a high degree of heterogeneity in terms of architecture, modality, setting, input and output data, functionalities, type of integration, and intended user group, which can pose limitations in terms of the generalizability of our findings. On a similar note, the absence of an organized framework outlining all value areas required the research team to develop their own set of value areas building on existing classification frameworks from the CDSS literature, by adding common value areas identified in the reviewed literature. Although this approach may also introduce a possible risk of bias, the potential impact was minimized through expert review and validation. Another limitation was the lack of granularity provided in the publications regarding the factors contributing to the limited success of certain CDSS implementations. While some authors mentioned factors such as suboptimal design, low adoption rate, organizational challenges, and issues with CDSS alerts, more detailed insights would greatly benefit future CDSS development.

### Conclusions

The utilization of CDSSs varied globally, with substantial application in the United States and within the domain of chronic cardio-renal-metabolic diseases. Overall, both integrated and stand-alone CDSSs have been successful in providing benefits to HCPs, regardless of the integration type. Our findings suggest that CDSSs have an important role in quality assurance in patient care. Flagging and treatment recommendation features are commonly used in CDSSs to improve patient care, while other features, tailored to specific requirements, collectively contribute to the efficiency of health care delivery. Due to a lack of granularity, as well as extensive heterogeneity in the evidence available, there remains uncertainty around the role of specific factors contributing to the success of CDSS. There is a need for real-world evidence from longitudinal studies to identify the challenges of the adoption and deployment of these systems.

## Appendix

Multimedia Appendix 1 Value areas.

Multimedia Appendix 2 Supplementary materials.

Multimedia Appendix 3 Features.

Multimedia Appendix 4 Disease areas.

Multimedia Appendix 5 CDSS study characteristics. CDSS: clinical decision support system.

Multimedia Appendix 6 Features of CDSSs for all successful studies. CDSS: clinical decision support system.

## Abbreviations

CDSS: clinical decision support system
CVD: cardiovascular disease
EMR: electronic medical record
HbA1c: hemoglobin A1c
HCP: health care professional
RCT: randomized controlled trial
TLR: targeted literature review
