# Supplementary material for: Benefits of Clinical Decision Support Systems for the Management of Noncommunicable Chronic Diseases: Targeted Literature Review
Source: Interact J Med Res. 2024 Nov 27;13:e58036. doi: 10.2196/58036 (PMC11635333; doi:10.2196/58036)
Supplement: Multimedia Appendix 1 [file ijmr_v13i1e58036_app1.docx]

A value area represents distinct categories where CDSSs can assist HCPs and end users to improve healthcare as well as to help understand the acceptability of these systems.

Value areas were classified in the TLR as follows:

- Quality Assurance: Processes in CDSS to ensure accuracy, reliability, and adherence to clinical standards contributing to safe and effective care.
- Clinical Benefit: CDSS improves clinical decision-making by providing guideline-based recommendations, improving diagnostic accuracy, and optimizing treatment choices thereby contributing to better clinical outcomes.
- User Satisfaction: Refers to the acceptability of CDSS and how satisfied HCPs or other end-users are with CDSS and its impact on their clinical practice.
- Guideline Adherence: Refers to a CDSS’s ability to help HCPs follow evidence-based clinical guidelines and best practices in patient care.
- Workflow Improvement: Supporting the HCPs in making the clinical workflows more streamlined, efficient, consistent, and patient-centered.
- Patient Behavior/Self-management: CDSS tools supports patients’ active involvement in managing their own health and wellbeing.
- Patient Safety and Risk: CDSS tools contribute to patient safety and risk management by providing evidence-based recommendations and reducing the likelihood of safety risks.
- Educational Aspects: Designed to enhance the knowledge and understanding related to clinical decision-making, which could contribute to improving patient care.

Financial Aspects: Economic implications associated with the implementation and utilization of CDSS tools within healthcare organizations.
