# Supplementary material for: Benefits of Clinical Decision Support Systems for the Management of Noncommunicable Chronic Diseases: Targeted Literature Review
Source: Interact J Med Res. 2024 Nov 27;13:e58036. doi: 10.2196/58036 (PMC11635333; doi:10.2196/58036)
Supplement: Multimedia Appendix 2 [file ijmr_v13i1e58036_app2.docx]

**
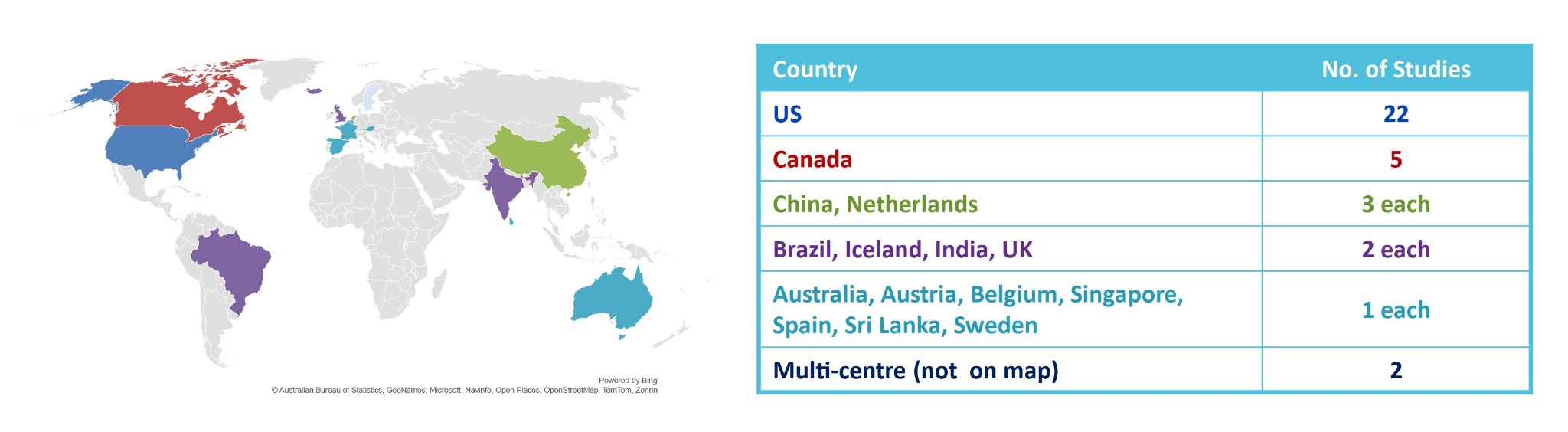
**

**Figure S1.** Evidence overview across geographies.


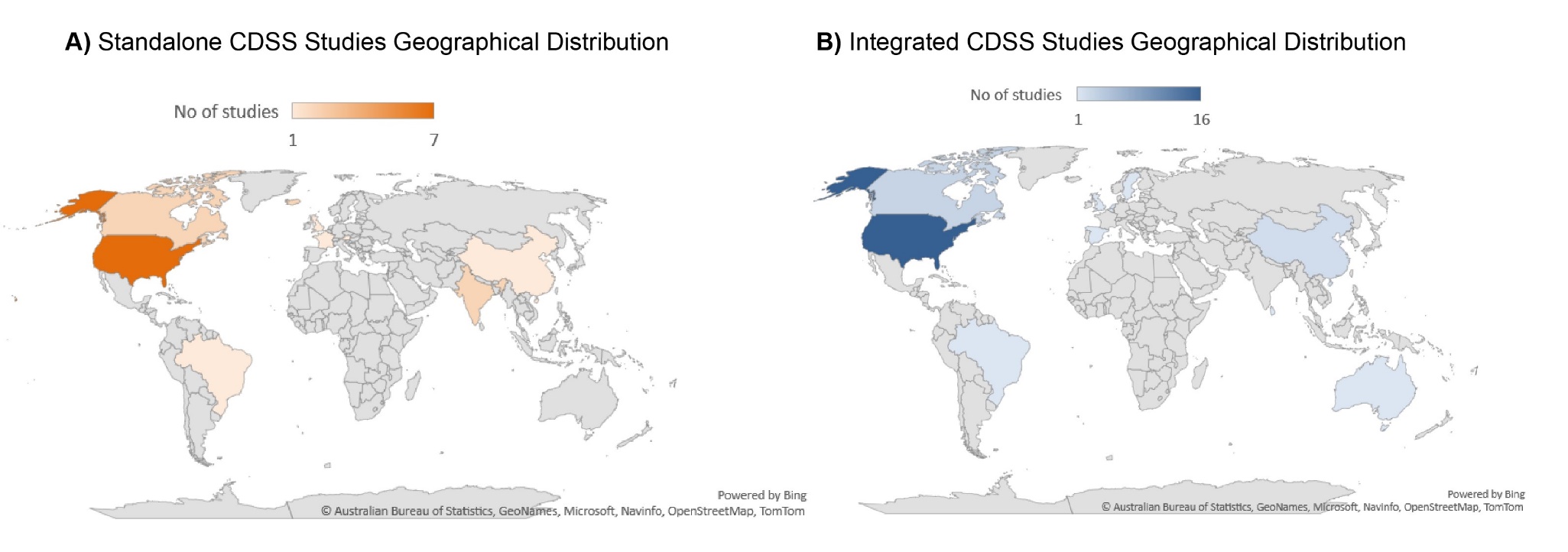


**Figure S2.** Geographical distribution of (A) standalone and (B) integrated studies.

**
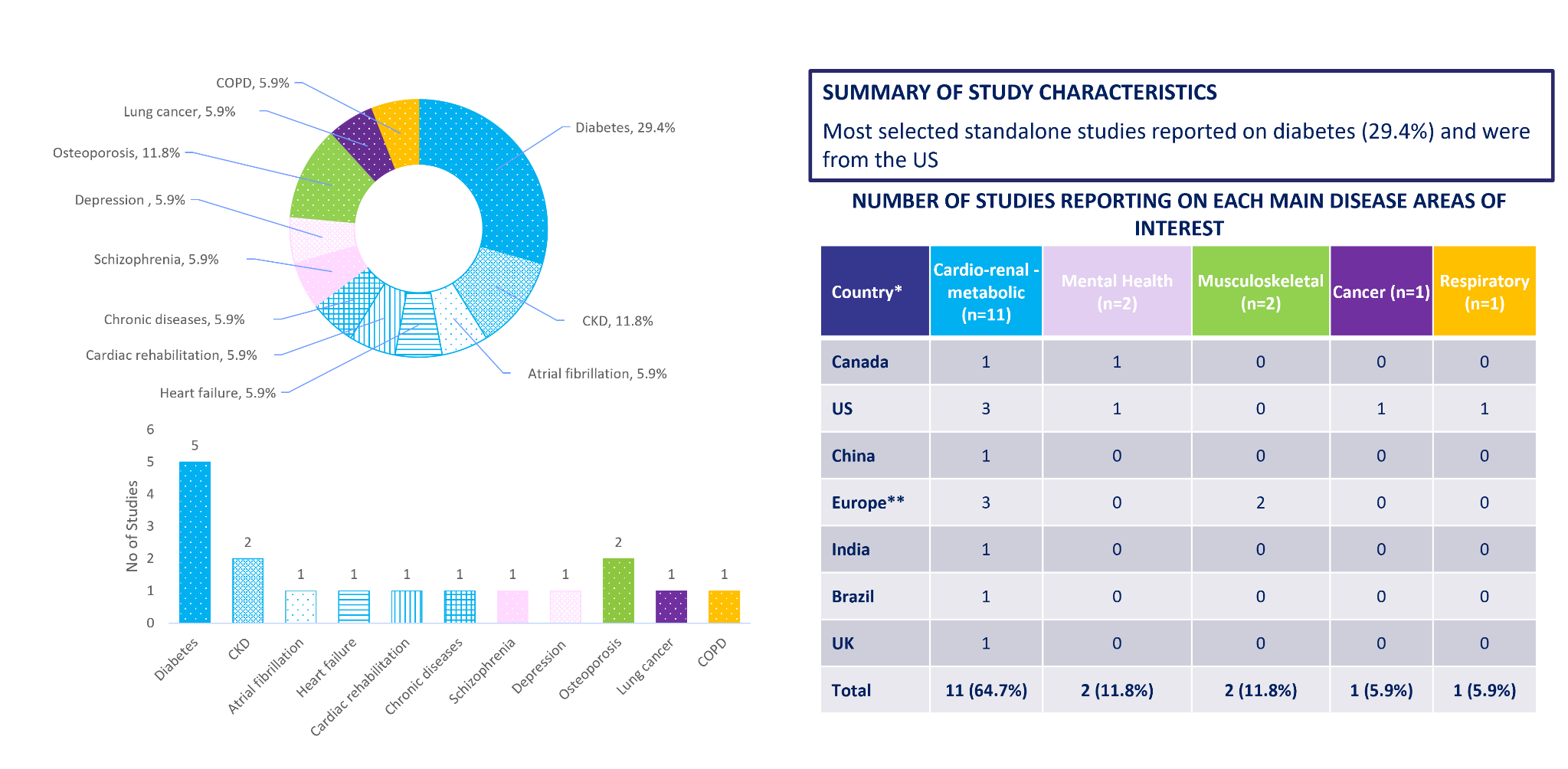
Figure S3.** Overview of standalone CDSS studies by target disease area. * Two studies were multi-country 1) Austria, Germany, Italy, and the United Kingdom 2) India and Pakistan. ** Europe= Austria, Sweden, Iceland, Belgium, Spain, Netherlands. Abbreviation: CDSS, clinical decision support system; CKD, chronic kidney disease; COPD, chronic obstructive pulmonary disease; UK, United Kingdom; US, United States


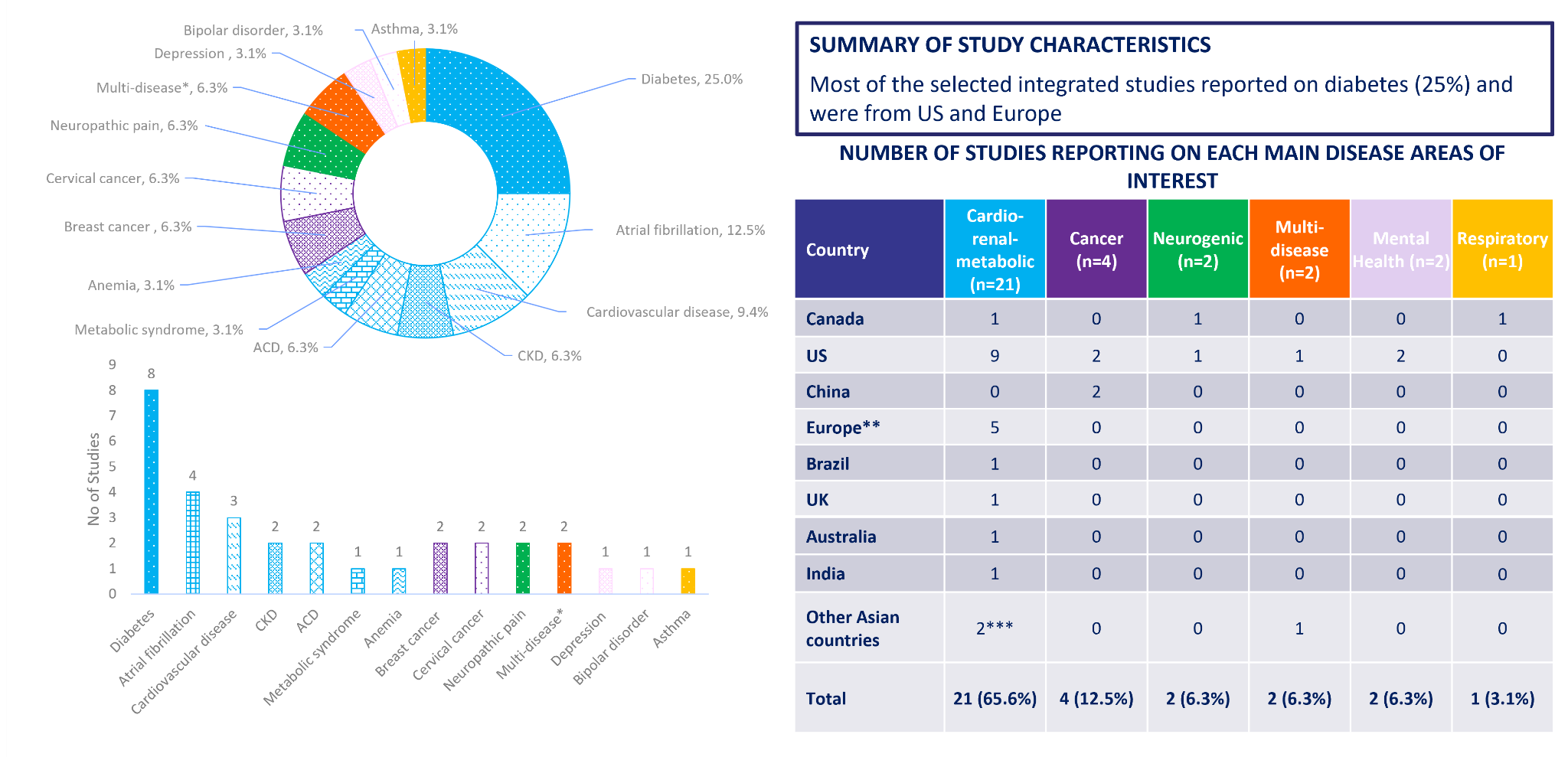
**Figure S4.** Overview of integrated CDSS studies by target disease area. *Multidisease: included Cardiorenal, Respiratory and Mental health.

** Europe= Austria, Sweden, Iceland, Belgium, Spain, Netherlands and a multicentre study in Austria, Germany, Italy, and the United Kingdom *** Includes one study of India and Pakistan. Abbreviation: CDSS, clinical decision support system; CKD, chronic kidney disease; ACD, atherosclerotic cardiovascular disease; UK, United Kingdom; US, United States

**Search Strategies**

**Table S1.** Ovid Algorithm—Embase

| Search Number | Search Terms |
| --- | --- |
| 1 | clinical decision support system/ |
| 2 | (decision support adj2 (clinical$ or system$ or tool$ or program$ or aid$)).ti,ab. |
| 3 | 1 or 2 |
| 4 | practice guideline/ or clinical protocol/ |
| 5 | (guideline$ or protocol$).ti,ab. |
| 6 | 4 or 5 |
| 7 | 3 and 6 |
| 8 | 7 not ((exp animal/ or nonhuman/) not exp human/) |
| 9 | (case report or case series or woman or man or child or adolescent or female or male or boy or girl or infant).ti. |
| 10 | case reports/ or case study/ or case report$.jx. or case report$.jw. |
| 11 | (Ephemera or "Introductory Journal Article" or News or "Newspaper Article" or Editorial or Comment or Overall).pt. or in vitro Techniques/ or in vitro study/ or (commentary or editorial or comment or letter or mice or rat or mouse or animal or murine).ti. |
| 12 | review.pt. not (systematic or (meta and analy*) or ((indirect or mixed) and treatment comparison)).ti,ab. |
| 13 | model.ti,ab. |
| 14 | or/9-13 |
| 15 | 8 not 14 |
| 16 | limit 15 to (article or article in press) |
| 17 | limit 15 to (conference abstract and yr="2020-current") |
| 18 | 16 or 17 |
| 19 | limit 18 to yr="2012-current" |

**Table S2.** Ovid Algorithm—Medline

| Search Number | Search Terms |
| --- | --- |
| 1 | decision support systems, clinical/ |
| 2 | (decision support adj2 (clinical$ or system$ or tool$ or program$ or aid$)).ti,ab. |
| 3 | 1 or 2 |
| 4 | practice guideline/ or clinical protocols/ or Guidelines as Topic/ |
| 5 | (guideline$ or protocol$).ti,ab. |
| 6 | 4 or 5 |
| 7 | 3 and 6 |
| 8 | 7 not (animals/ not humans/) |
| 9 | (case report or case series or woman or man or child or adolescent or female or male or boy or girl or infant).ti. |
| 10 | case reports/ or case study/ or case report$.jw. |
| 11 | (Ephemera or "Introductory Journal Article" or News or "Newspaper Article" or Editorial or Comment or Overall).pt. or in vitro Techniques/ or in vitro study/ or (commentary or editorial or comment or letter or mice or rat or mouse or animal or murine).ti. |
| 12 | review.pt. not (systematic or (meta and analy*) or ((indirect or mixed) and treatment comparison)).ti,ab. |
| 13 | model.ti,ab. |
| 14 | or/9-13 |
| 15 | 8 not 14 |
| 16 | limit 15 to yr="2012-current" |

**Table S3.** Ovid Algorithm—Cochrane CENTRAL Register of Controlled Trials

| Search Number | Search Terms |
| --- | --- |
| 1 | (decision support adj2 (clinical$ or system$ or tool$ or program$ or aid$)).ti,ab. |
| 2 | (guideline$ or protocol$).ti,ab. |
| 3 | 1 and 2 |
| 4 | (EUCTR$ or NCT$ or ICTRP$ or CTRI$ or ISRCTN$ or chictr$ or actrn$ or IRCT$ or NTR$).tn. |
| 5 | (journal conference abstract or journal conference paper or "journal conference review").pt. |
| 6 | model$.ti,ab. |
| 7 | 3 not (4 or 5 or 6) |
| 8 | limit 7 to yr="2012 -Current" |

**Table S4.** Ovid Algorithm—Cochrane Database of Systematic Reviews

| Search Number | Search Terms |
| --- | --- |
| 1 | (decision support adj2 (clinical$ or system$ or tool$ or program$ or aid$)).ti,ab. |
| 2 | (guideline$ or protocol$).ti,ab. |
| 3 | 1 and 2 |
| 4 | limit 3 to yr="2012 -Current" |

|  | **Education** | **Data export** | **Flagging** | **Risk level estimation** | **Tx** |
| --- | --- | --- | --- | --- | --- |
| **Clinical benefits (n=2)** | 1/2 | 1/2 | 1/2 | 0 | 2/2 |
| **Patient safety and risk management (n=1)** | 0 | 0 | 0 | 0 | 1/1 |
| **Educational aspects (n=1)** | 1/1 | 0 | 1/1 | 1/1 | 1/1 |
| **User satisfaction (n=1)** | 0 | 0 | 1/1 | 0 | 1/1 |
| **Quality assurance (n=3)** | 1/3 | 1/3 | 2/3 | 0 | 3/3 |
| **Guideline adherence (n=2)** | 0 | 0 | 1/2 | 0 | 1/2 |
| **Patient behavior/self-management (n=1)** | 1/1 | 0 | 1/1 | 1/1 | 1/1 |

**Figure S5.** Heatmaps of Unsuccessful All. Tx, treatment

The heatmap is a graphical representation of the distribution of CDSS features across value areas. The heatmap data matrix is interpreted as (x/y) *100% where “x” represents the frequency of CDSS features reported across studies and “y” represents the total number of studies reporting on the specific value area. The heatmap uses color mapping with grey assigned to no values, yellow assigned to low values, and dark green assigned to highest values. Heatmap legend, in order of increasing color intensity to indicate how frequently features were reported: yellow gradient=<30%, <45%; green gradient=<60%, <90%, ≥90%; grey=0; dec: decision; rec: recommendation

**Table S5.** Examples of categories for tagging.

| Disease area: Abdominal-biliary infection, Acid suppression; Acute coronary syndrome; Acute diarrhea; Acute sinusitis; Acute sinusitis; Antibiotic resistance; Asthma; Atherosclerotic cardiovascular disease; Atrial fibrillation; Blood transfusion; Cardiovascular disease; Cardiovascular disease; Chronic kidney disease; Chronic obstructive pulmonary disease; Clostridium difficile infection; Coronary heart disease; Coronary syndrome; Depression; Dermatology and obstetrics; Diabetes; Elderly patients with multimorbidity; Elective surgery; Emergency medicine; Fractures (rib); Heart failure; Hepatitis; Infectious disease; Lower back pain; Lung Cancer; Multiple myeloma; Neonatal care; Neuropathic pain; Nonpurulent cellulitis; Nutrition orders; Oncology; Palliative care; Pharmacy; Pneumonia; Polypharmacy; Preeclampsia; Prescribing; Preventative care; Prevention of delirium (elderly); Sepsis; Sickle cell disease; Stage 3 or 4 cancers; Stroke; Tobacco use screening in dental clinics; Trauma management; Type 1 diabetes; Type 2 diabetes; Urinary tract infection; Venous Thromboembolism |
| --- |
| Features that impact successful implementation: Standalone or EMR-integrated CDSS; computer or mobile device |
| Value areas included: Clinical benefits, Workflow improvements, Educational aspects, Guideline adherence, Quality assurance |
| Methods used to integrate/implement CDSS within already established EHR/EMR: Reported or not reported |
| User groups: Physicians, nurses, clinicians, specialists |
| Investigational or marketed CDSS |
| Main function of CDSS in disease management if pertaining to treatment management only, treatment management and other combined diagnostics |
| Study design: RCT or observational studies |
| Geography: Specify country where study was conducted |

**Table S6.** Data extraction elements

| Study Objective |
| --- |
| Country |
| Study design |
| CDSS tool |
| CDSS function (System Output) |
| Type of data entering CDSS |
| Investigational or marketed |
| Disease area |
| Study setting |
| Primary Outcomes |
| Secondary Outcomes |
| User group |
| Value areas of CDSS   - - Clinical benefits   - Patient Safety and Risk Management   - Workflow improvements   - Educational aspects   - User satisfaction   - Quality assurance   - Guideline adherence   - Patient Behavior /Self-management   - Other value areas, if reported |
| Features and feature categories of CDSS |
| Automatic vs. Manual entry |
| Features of CDSS that impact the successful adoption |
| Modality |
| Stand-alone or integrated |
| Methods used to integrate/implement CDSSs within already established EMRs |
| Did the study achieve its objectives |
| Author conclusions |
| Limitation of the study |
