# Supplementary material for: Benefits of Clinical Decision Support Systems for the Management of Noncommunicable Chronic Diseases: Targeted Literature Review
Source: Interact J Med Res. 2024 Nov 27;13:e58036. doi: 10.2196/58036 (PMC11635333; doi:10.2196/58036)
Supplement: Multimedia Appendix 3 [file ijmr_v13i1e58036_app3.docx]

Features refer to specific functionalities that a CDSS provides to support clinical decision making. CDSS features were classified in the TLR as follows:

- Treatment recommendations: Facilitates pharmacotherapy by suggesting prescribing medications, offering dosage recommendations, checking for drug interactions, and ensuring adherence to guidelines.
- Flagging: Alerts HCPs to important information that may require attention, ensuring timely intervention and follow-up such as abnormal test results, missed follow-up appointments, and safety monitoring.
- Risk-level estimation: Estimates a patient’s risk level for certain conditions or events.
- Diagnosis: Assists in the diagnostic process by providing relevant information, suggesting potential diagnoses, and offering decision support based on patient data.
- Education: Offers educational resources, guidelines, and references to HCPs to support continuous learning.
- Data Export: Supports and facilitates the export of data, reports, and other documentation for analysis.
- Monitoring: Facilitates simultaneous monitoring of multiple parameters in patients. This continuous monitoring informs HCPs on potential issues with patient’s health, tracks the progress of treatments, and makes adjustments as needed.
- Shared decision making: Facilitates collaborative and shared decision-making by providing relevant information to both HCPs and patients.
- Audit: Supports auditing capabilities to track and review clinical decisions and actions taken by HCPs. Auditing features help ensure accountability, quality assurance, and adherence to guidelines.
- Referral: Supports with referral recommendations to specialists based on the analysis of patient data and the complexity of the case.
- Screening: Includes screening tools and reminders for preventive care and routine screenings.
- Patient self-management: Supports patients in managing their own health by providing a comprehensive view of their health data, educational materials, and resources to make informed decisions on self-management.
- Safety: Contributes to patient safety by offering alerts for potential adverse drug reactions, safety concerns, and evidence-based recommendations.
- Optional scope: Includes features that can be tailored based on specific needs and preferences of HCPs.
