# Supplementary material for: Benefits of Clinical Decision Support Systems for the Management of Noncommunicable Chronic Diseases: Targeted Literature Review
Source: Interact J Med Res. 2024 Nov 27;13:e58036. doi: 10.2196/58036 (PMC11635333; doi:10.2196/58036)
Supplement: Multimedia Appendix 4 [file ijmr_v13i1e58036_app4.docx]

| Disease areas of CDSS tools in the studies | TLR disease area classification |
| --- | --- |
| Cervical cancer | Cancer |
| Breast cancer (of ductal, lobular, metaplastic, or mixed histology) |  |
| Lung cancer |  |
| Anemia in chronic kidney disease  Atherosclerotic cardiovascular disease | Cardiorenal |
| Chronic kidney disease |  |
| Atrial fibrillation |  |
| Type 2 diabetes |  |
| Heart failure |  |
| Hyperlipidemia |  |
| Hypercholesterolemia  Hypertension |  |
| Metabolic syndrome |  |
| Chronic diseases |  |
| Cardiovascular disease |  |
| Cardiac rehabilitation |  |
| Bipolar disorder | Mental Health |
| Major depressive disorder |  |
| Schizophrenia,  schizophreniform disorder,  Psychotic disorder not otherwise specified |  |
| Depression |  |
| Osteoporosis | Musculoskeletal |
| Neuropathic pain | Neurological |
| Painful polyneuropathy |  |
| Chronic obstructive pulmonary disease | Respiratory |
| Asthma |  |
