# Supplementary material for: Benefits of Clinical Decision Support Systems for the Management of Noncommunicable Chronic Diseases: Targeted Literature Review
Source: Interact J Med Res. 2024 Nov 27;13:e58036. doi: 10.2196/58036 (PMC11635333; doi:10.2196/58036)
Supplement: Multimedia Appendix 5 [file ijmr_v13i1e58036_app5.docx]

**Multimedia Appendix 5: CDSS Study Characteristics**

**Table S1. CDSS study characteristics**

| **First author, year** | **Country** | **CDSS name or descriptor** | **Disease area** | **Study design** | **Value Areas** | **Did the study achieve its objectives?** | Deemed Successful/Unsuccessful in the TLR |
| --- | --- | --- | --- | --- | --- | --- | --- |
| Adusumalli, 2021 [1] | US | EMR-based CDSS tool | Cardio-renal-metabolic (ASCVD) | Cluster-randomized trial | User satisfaction  Guideline adherence  Quality assurance | No, as the passive and active choice interventions embedded within the EMR did not change statin prescribing among cardiologists although, this CDSS tool benefitted those diagnosed with ASCVD to an extent. | Unsuccessful CDSS |
| Ahmed, 2014 [2] | Canada | ADSS linked to provincial administrative database | Respiratory (asthma) | Retrospective cohort study | Quality assurance | Yes, as it was able to identify individuals who might benefit from a medication review. | Successful CDSS |
| Ajay, 2016 [3] | India | “mPower Heart Project” | Cardio-renal-metabolic (hypertension and DM) | Single-arm trial, open-label | Clinical benefits  Quality assurance | Yes, as the study demonstrated the feasibility of nurse-facilitated, mobile phone–based decision support software system-enabled intervention for hypertension and diabetes mellitus care. | Successful CDSS |
| Alhodaib, 2020 [4] | UK | Mobile CDSS app | Cardio-renal-metabolic (CKD in patients with DM) | RCT and semi structured interview | User satisfaction  Guideline adherence | Partially yes, as the application was successfully developed and the user group reported high level of satisfaction; however, the application was not found to improve the adherence to the guidelines | Partially Successful CDSS |
| Ali, 2016 [5] | India and Pakistan | “mWellcare”, an mHealth system, integrated with EMRs | Cardio-renal-metabolic (T2DM) | RCT, open-label | Clinical benefits  Patient safety and risk  Quality assurance Patient Behavior /Self-management | Yes, the multi-component quality improvement, including the CDSS, improved the rates of participants achieving diabetes care goals. | Successful CDSS |
| Cohen, 2020 [6] | US | Computerized best practice alert system, integrated with EMRs | Cardio-renal-metabolic (metabolic syndrome) | Retrospective chart review | Quality assurance | Yes, the active best practice alert system was an effective tool in increasing the metabolic monitoring in patients taking second-generation antipsychotics. | Successful CDSS |
| Comin, 2017 [7] | Spain | Complex system that integrated eCPGs with EMRs | Cardio-renal-metabolic (hypertension, hypercholesterolemia, and T2DM) | Pre/post RCT | User satisfaction  Clinical benefits | Yes, the patients attended by users of eCPGs had better control and follow-up of cardiovascular risk factors than those attended by eCPG non-users. | Successful CDSS |
| Conway, 2018 [8] | UK | CDSS based on the EBMeDS system and integrated with EMRs | Cardio-renal-metabolic (T2DM) | Case-control study | Clinical benefits  User satisfaction  Guideline adherence  Workflow improvements | Yes, the CDSS was well received; and was associated with improved efficiencies in working practices, along with noticeable improvements in guideline adherence. | Successful CDSS |
| Cox, 2020 [9] | Canada | Computerized CDSS | Cardio-renal-metabolic (AF) | Cluster randomized trial | Clinical benefits  Patient Safety and Risk  Quality assurance | No, as the CDSS tool did not have an impact on AF management related outcomes. Although usual care participants were 16% more likely to have more than one primary outcome event than CDSS arm, the observed difference was not significant. | Unsuccessful CDSS |
| Ennis, 2015 [10] | US | Guideline-based CDSS | Cardio-renal-metabolic (CKD) | Matched cohort study | Quality assurance  Guideline adherence | Yes, the use of this automated laboratory based CDSS was found to have improved physician adherence to guidelines with respect to timely monitoring of CKD. | Successful CDSS |
| Garcia, 2013 [11] | Brazil | Guideline-based CDSS, integrated with EMRs | Cardio-renal-metabolic (anemia in CKD) | Retrospective cohort study | Quality assurance | Yes, some good initial results were obtained so far (added along with its beneficial feature of having incorporated generic method and applicability to any type of guideline) on this approach which is still under research. | Successful CDSS |
| Gill, 2012 [12] | US | EMR-based CDSS tool | Mental health (depression) | Prospective cohort study | User satisfaction  Workflow improvements  Educational aspects  User satisfaction  Quality assurance | Partially yes, the CDSS was extensively used and perceived as very helpful in assessment of patients’ symptoms but not in provider education. | Partially Successful CDSS |
| Gill, 2012 [13] | US | EMR-based CDSS tool | Mental health (bipolar disorder) | Nonrandomized, controlled trial | Quality assurance | Yes, this EMR-based tool was found to be useful in screening and management of bipolar disorder in patients with depression. | Successful CDSS |
| Gill, 2019 [14] | US | Point of care CDSS integrated with EMRs | Cardio-renal-metabolic (T2DM)^a^ | Prospective cohort study | Clinical benefits  Quality assurance  User satisfaction | Yes, the use of this CDSS resulted in small but statistically significant reductions in both HbA1C and LDL-C. There were also statistically significant, clinically meaningful increases in the odds of achieving personalized HbA1C and LDL-C goals in the CDSS group. | Successful CDSS |
| Gudmundsson, 2019 [15] | Iceland | “OPAD” based on clinically relevant parameters | Musculoskeletal (osteoporosis) | Retrospective cohort study | Quality assurance | Yes, recommendations  given by OPAD were consistent with expert opinion and existing guidelines. | Successful CDSS |
| Guenter, 2019 [16] | Canada | “McMaster Pain Assistant” CDSS, integrated with EMRs | Neurological (neuropathic pain) | Quasi-experimental, pre/post study | Guideline adherence  User satisfaction | Partially yes, as there was a minimal utilization of the CDSS as a decision support tool and as a data entry tool. But it was highly utilized for reference information. | Partially Successful CDSS |
| Gunathilake, 2013 [17] | Sri Lanka | System of EMR with decision support prompts | Cardio-renal-metabolic (T2DM) | Case-control study | Clinical benefits  Quality assurance | Yes, the use of this decision support system showed benefits in adherence to clinical care pathways and thereby achieving significant improvement in glycemic control. | Successful CDSS |
| Halldorsson, 2015 [18] | Iceland | “OPAD” CDSS based on clinically relevant parameters | Musculoskeletal (osteoporosis) | Cross-sectional study | Quality assurance | Yes, OPAD was accurate in respect to fracture risk probability evaluation and might presumptively be cost effective in fracture liaison services. | Successful CDSS |
| Hendriks, 2019 [19] | Netherlands | “CardioConsult AF®”, integrated with EMRs | Cardio-renal-metabolic (AF) | RCT, open-label | Clinical benefits | Yes, the integrated specialized AF-Clinic reduced all-cause mortality compared with usual care. | Successful CDSS |
| Heselmans, 2020 [20] | Belgium | EBMeDS system, the CCDS system of EBPNet, a national computerized point-of-care information service integrated with EMRs | Cardio-renal-metabolic (T2DM) | Cluster randomized trial | Clinical benefits | No, the EBMeDS system did not improve diabetes care in Belgian primary care. | Unsuccessful CDSS |
| Huang, 2016 [21] | US | “Personal DC”, a personalized Web-based CDSS | Cardio-renal-metabolic (T2DM) | RCT | Quality assurance  Educational aspects  User satisfaction | Yes, the CDSS significantly reduced patients’ informed subscale of decisional conflict scores. Other findings were not statistically significant but promising. | Successful CDSS |
| Karlsson, 2018 [22] | Sweden | EMR-based CDSS tool | Cardio-renal-metabolic (AF) | Cluster randomized trial | Clinical benefits  Quality assurance  Patient safety and risk | Yes, the CDSS could increase guideline adherence for anticoagulant therapy in patients with AF. | Successful CDSS |
| Kropf, 2014 [23] | Austria | Algorithm based CDSS | Cardio-renal-metabolic (HF) | Cross-sectional study | Quality assurance | Yes, since the goal of the CDSS tool developed (i.e., to increase the dose of HF medication to reach the target dose) was aligned with the findings from a retrospective analysis with the existing telemonitoring datasets which indicated that the current rule implementation tends to advice to increase the dose of HF medication. | Successful CDSS |
| Litvin, 2016 [24] | US | EMR-based CDSS tool | Cardio-renal-metabolic (CKD) | Observational longitudinal study | Workflow improvements  Educational aspects  User satisfaction  Quality assurance | Partially yes, as the intervention resulted in a dramatic increase in the number of patients at risk for and with CKD who received testing for albuminuria.  Also, the intervention did not result in significant changes in performance on the other clinical quality measures. | Partially Successful CDSS |
| Lobach, 2016 [25] | US | “SAMI-L”, a web-based CDSS tool | Cancer (lung cancer) | Retrospective study and structured interviews, and questionnaires | Workflow improvements  User satisfaction  Patient behavior /self-management | Yes, both patients and HCPs found the CDSS to be usable and acceptable in preclinical and clinical settings. | Successful CDSS |
| Luo, 2019 [26] | China | CDSS software developed according to the Chinese Diabetes Society guideline | Cardio-renal-metabolic (T2DM) | Prospective cohort study | Quality assurance | Yes, an improvement in standard care including statin and aspirin usage was noticed. | Successful CDSS |
| Marcolino, 2021 [27] | Brazil | “HealthRise” | Cardio-renal-metabolic (hypertension and DM) | Single-arm, longitudinal, observational study | User satisfaction | Yes, the CDSS was developed and implemented in primary-care units, with good user satisfaction. | Successful CDSS |
| McKie, 2020 [28] | US | “MayoExpertAdvisor”, an EMR-based CDSS tool | Cardio-renal-metabolic (HF, hyperlipidemia, and AF) | Cluster randomized trial | Guideline adherence  Workflow improvements  Quality assurance | Partially yes, as the CDSS improved the adherence to guideline-recommended treatment for HF but not for AF or hyperlipidemia | Partially Successful CDSS |
| Morganroth, 2016 [29] | US | “CareManager” | Respiratory (COPD) | RCT and quasi-experimental study | Clinical benefit  Guideline adherence | Yes, as the RCT suggested the intervention group as having a higher number of guidelines directed interventions per patient, even though there was no observable favor regarding clinical outcomes. Similarly, the quasi-experimental study reported only fewer COPD exacerbations and an increased adherence to guideline recommendations after the implementation of CareManager | Successful CDSS |
| O’Connor, 2011 [30] | US | “Diabetes Wizard”, an EMR-based CDSS tool | Cardio-renal-metabolic (T2DM) | RCT | Clinical benefits  Quality assurance  User satisfaction | Yes, the CDSS has significantly improved the glucose control and some aspects of blood pressure control in adults with T2DM. | Successful CDSS |
| Orchard, 2020 [31] | Australia | EMR-based electronic CDSS app | Cardio-renal-metabolic (AF) | Cross-sectional study and cost-effectiveness analysis | Quality Assurance  Financial aspects | Yes, the unique suite of integrated, customized eHealth tools was able to support all stages of AF screening and treatment in general practice. These eHealth tools and regular data reports were thus well-accepted. | Successful CDSS |
| Popescu, 2021 [32] | Canada | AI-powered “Aifred” CDSS tool | Mental health (MDD) | Single-arm, longitudinal, observational study | Workflow improvements  Quality assurance  Patient safety and risk  User satisfaction  Patient behavior /self-management | Yes, as the appointment length did not significantly increase after the introduction of the tool; it also resulted in improved patient-clinician relationship for some patients, and patients and clinicians both engaged with the app in a consistent manner that supports clinical feasibility. | Successful CDSS |
| Prabhakaran, 2019 [33] | India | “mWellcare”, an mHealth system integrated with EMRs | Multidisease – cardio-renal-metabolic, respiratory and mental health (T2DM, hypertension, tobacco and alcohol use, and depression) | Cluster randomized trial | Clinical benefits  Quality assurance  Patient behavior /self-management | No, as there was no incremental benefit of mWellcare over enhanced usual care in the management of the studied chronic conditions. | Unsuccessful CDSS |
| Regan, 2017 [34] | US | CDSS tool based on NKF-KDOQI guidelines and integrated with EMRs | Cardio-renal-metabolic (CKD) | Pre/post surveys | Clinical benefits  Educational aspects  User satisfaction | Yes, as the primary-care providers increased their overall knowledge of CKD stages, diagnostic criteria, and the significance of the albumin/creatinine result value in the detection of underlying renal pathologic abnormalities. Also, there was an improvement in referring patients based on eGFR and albuminuria parameters. | Successful CDSS |
| Reilly, 2012 [35] | Canada | “COMPETE II”, a web-based tool, interfaced with the patient’s EMR | Cardio-renal-metabolic (T2DM) | RCT | Clinical benefits  Financial implications | Yes, the web-based prototype decision support system slightly improved short-term risk factors and the model predicted moderate improvements in long-term health outcomes. | Successful CDSS |
| Reynolds, 2020 [36] | US | CDSS that used a best practice alert linked to Smartset and integrated with EMRs | Neurological (painful polyneuropathy) | RCT | Quality assurance  Guideline adherence | No, the proposed CDSS was unsuccessful, both in its use and in altering the prescribing patterns of guideline recommended medications. | Unsuccessful CDSS |
| Rieckert, 2020 [37] | Austria, Germany, Italy, and the UK | “PRIMA-eDS” | Cardio-renal-metabolic (chronic diseases) | Cluster randomized trial | Clinical benefits  Patient safety and risk  Quality assurance | Partially yes, this computerized decision support tool for comprehensive drug review of elderly people with polypharmacy showed no conclusive  effects on the composite of unplanned hospital admission or death by 24 months. Nonetheless, a reduction in drugs was achieved without detriment to patient outcomes. | Partially Successful CDSS |
| Robinson, 2018 [38] | US | COMPASS, a NAVIGATE-developed computerized CDSS tool | Mental health (schizophrenia, schizoaffective disorder, schizophreniform disorder, brief psychotic disorder, or psychotic disorder not otherwise specified | Cluster randomized trial | Clinical benefits  Patient safety and risk  Quality assurance  Guideline adherence | Yes, the study findings supported the sustained feasibility and acceptability of the NAVIGATE treatment model in comparison with usual care. | Successful CDSS |
| Rodbard, 2012 [39] | US | “Accu-Chek 360°”, an automated DST | Cardio-renal-metabolic (T2DM) | RCT | User satisfaction  Quality assurance  Workflow improvements | Yes, use of structured SMBG, combined with the DST, the educational DVD, or both, enhanced clinicians’ ability to correctly identify significant glycemic patterns and make  appropriate therapeutic decisions to address those patterns. | Successful CDSS |
| Rossom, 2022 [40] | US | EMR-based CDSS tool | Mental health (bipolar disorder, schizoaffective disorder/schizophrenia and CVD) | Cluster randomized trial | Clinical benefits | Yes, this CDS intervention resulted in a rate of change in total modifiable CV risk that was 4% lower among intervention patients as compared with the control patients. | Successful CDSS |
| Scheitel, 2017 [41] | US | “MEA”, an EMR-based CDSS tool | Cardio-renal-metabolic (ASCVD) | Pre-post analysis | Workflow improvements  Quality assurance  Guideline adherence  User satisfaction | Yes, the CDSS could greatly improve the efficiency and accuracy of individualized treatment recommendations and had the potential to increase guideline compliance. | Successful CDSS |
| Sim, 2017 [42] | Singapore | “Diabetes Dashboard”, an EMR-based CDSS tool | Cardio-renal-metabolic (T2DM) | RCT | Workflow improvements  Quality assurance  User satisfaction | Yes, the CDSS developed could improve the management of diabetes. | Successful CDSS |
| Sperl-Hillen, 2018 [43] | US | Web-based, point-of-care CDSS system, integrated within the EMR and primary-care workflow | Cardio-renal-metabolic (CVD) | RCT | Clinical benefits  User satisfaction  Quality assurance | Yes, the CDSS could significantly reduce CV risk in targeted primary-care patients. | Successful CDSS |
| Vromen, 2021 [44] | Netherlands | Web-based CDSS tool with an interactive system | Cardio-renal-metabolic (cardiac rehabilitation) | Pseudo-randomized, open-label study | Guideline adherence | No, the CDSS did not effectuate changes in ECR prescribing. | Unsuccessful CDSS |
| Wagholikar, 2012 [45] | US | CDSS prototype built on the drools platform and integrated with EMRs | Cancer (cervical cancer) | Retrospective cohort study | Quality assurance | Yes, a high accuracy of the CDSS was observed, and hence was considered as a suitable candidate for deployment in clinical practice. | Successful CDSS |
| Wagholikar, 2013 [46] | US | EMR-based, web application | Cancer (cervical cancer) | Retrospective cohort study | Quality assurance  Workflow improvements | Partially yes, the CDSS had a high level of accuracy, with the potential to improve providers’ recommendations especially in the high utility areas of the guidelines and could thereby significantly advance the quality of screening. However, the corrected CDSS was not tested with new cases, which would be of benefit to determine whether further discrepancies in recommendations need to be addressed. | Partially Successful CDSS |
| Wijtvliet, 2020 [47] | Netherlands | “CardioConsult AF®”, a guideline based CDSS software, integrated with EMRs | Cardio-renal-metabolic (AF) | RCT | Clinical benefits  Educational aspects  Guideline adherence  Behavioral change  Cost | No, nurse-led care did not significantly reduce the risk of cardiovascular death or hospital admission compared with usual-care. | Unsuccessful CDSS |
| Xu, 2019 [48] | China | “WfO”, an EMR-based advisory tool | Cancer (breast cancer) | Cross-sectional study | Quality assurance  Guideline adherence | Partially yes, additional research in different practice settings is needed to understand the tool’s scalability and its impact on treatment decisions and clinical and health services outcomes. | Partially Successful CDSS |
| Xu, 2020 [49] | China | “WfO”, an EMR-based tool | Cancer (breast cancer) | Cross-sectional study | Quality assurance  Guideline adherence | Yes, use of the CDSS tool had a significant impact on treatment decisions and adherence to NCCN guideline adherence in of HR-positive breast cancer. | Successful CDSS |

Notes:

^a^ Although the focus of the intervention was T2DM, the study did not exclude persons with type 1 or other diabetes as a distinction was not available in their International Classification of Diseases coding.

Abbreviations: ADSS, Asthma decision support system; AF, atrial fibrillation; AI, artificial intelligence; ASCVD, atherosclerotic cardiovascular disease; CCDS, computerized clinical decision support; CDSS, clinical decision support system; CKD, chronic kidney disease; COMPETE, Computerization of Medical Practices for the Enhancement of Therapeutic Effectiveness; COPD, chronic obstructive pulmonary disease; CVD, cardiovascular; CVD, cardiovascular disease; DC, diabetes care; DM, diabetes mellitus; DST, decision support tool; EBMeDS, Evidence Based Medicine electronic Decision Support; eCPG, electronic version of clinical practice guidelines; ECR, exercise-based cardiac rehabilitation; eGFR, estimated glomerular filtration rate; EHR, electronic health record; EMR, electronic medical record; HbA1c, glycated hemoglobin; HCP, health care professional; HF, heart failure; HR, hormone receptor; LDL-C, low density lipoprotein-cholesterol; MDD, major depressive disorder; MEA, MayoExpertAdvisor; NCCN, National Comprehensive Cancer Network; NKF-KDOQI, National Kidney Foundation/Kidney Disease Outcome Quality Initiative; OPAD, Osteoporosis Adviser; PRIMA-eDS, polypharmacy in chronic diseases: reduction of inappropriate medication and adverse drug events in older populations by electronic decision support; RCT, randomized controlled trial; SAMI-L, Symptom Assessment and Management Intervention for Lung cancer; SMBG, self-monitoring of blood glucose; T2DM, type 2 diabetes mellitus; Tx, treatment recommendations; UK, United Kingdom; US, United States; WfO, Watson for Oncology

**References**

1. Adusumalli S, Westover JE, Jacoby DS, Small DS, VanZandbergen C, Chen J, Cavella AM, Pepe R, Rareshide CA, Snider CK. Effect of passive choice and active choice interventions in the electronic health record to cardiologists on statin prescribing: a cluster randomized clinical trial. JAMA Cardiol. 2021;6(1):40-8. doi: 10.1001/jamacardio.2020.4730.

2. Ahmed S, Tamblyn R, Winslade N. Using decision support for population tracking of adherence to recommended asthma guidelines. BMJ Open. 2014 Mar 4;4(3):e003759. PMID: 24595132. doi: 10.1136/bmjopen-2013-003759.

3. Ajay VS, Jindal D, Roy A, Venugopal V, Sharma R, Pawar A, Kinra S, Tandon N, Prabhakaran D. Development of a smartphone-enabled hypertension and diabetes mellitus management package to facilitate evidence-based care delivery in primary healthcare facilities in india: the mPower heart project. J Am Heart Assoc. 2016 Dec 21;5(12). PMID: 28003248. doi: 10.1161/jaha.116.004343.

4. Alhodaib HI, Antza C, Chandan JS, Hanif W, Sankaranarayanan S, Paul S, Sutcliffe P, Nirantharakumar K. Mobile clinical decision support system for the management of diabetic patients with kidney complications in UK primary care settings: mixed methods feasibility study. JMIR Diabetes. 2020 Nov 18;5(4):e19650. PMID: 33206055. doi: 10.2196/19650.

5. Ali MK, Singh K, Kondal D, Devarajan R, Patel SA, Shivashankar R, Ajay VS, Unnikrishnan A, Menon VU, Varthakavi PK. Effectiveness of a multicomponent quality improvement strategy to improve achievement of diabetes care goals: a randomized, controlled trial. Ann Intern Med. 2016;165(6):399-408.

6. Cohen S, Bostwick JR, Marshall VD, Kruse K, Dalack GW, Patel P. The effect of a computerized best practice alert system in an outpatient setting on metabolic monitoring in patients on second-generation antipsychotics. J Clin Pharm Ther. 2020 Dec;45(6):1398-404. PMID: 32767599. doi: 10.1111/jcpt.13236.

7. Comin E, Catalan-Ramos A, Iglesias-Rodal M, Grau M, Del Val JL, Consola A, Amado E, Pons A, Mata-Cases M, Franzi A, Ciurana R, Frigola E, Cos X, Davins J, Verdu-Rotellar JM. Impact of implementing electronic clinical practice guidelines for the diagnosis, control and treatment of cardiovascular risk factors: A pre-post controlled study. Aten Primaria. 2017 Aug-Sep;49(7):389-98. PMID: 28314542. doi: 10.1016/j.aprim.2016.11.007.

8. Conway N, Adamson KA, Cunningham SG, Emslie Smith A, Nyberg P, Smith BH, Wales A, Wake DJ. Decision support for diabetes in Scotland: implementation and evaluation of a clinical decision support system. J Diabetes Sci Technol. 2018 Mar;12(2):381-8. PMID: 28905658. doi: 10.1177/1932296817729489.

9. Cox JL, Parkash R, Foster GA, Xie F, MacKillop JH, Ciaccia A, Choudhri SH, Hamilton LM, Nemis-White JM, Thabane L. Integrated Management Program Advancing Community Treatment of Atrial Fibrillation (IMPACT-AF): A cluster randomized trial of a computerized clinical decision support tool. Am Heart J. 2020 Jun;224:35-46. PMID: 32302788. doi: 10.1016/j.ahj.2020.02.019.

10. Ennis J, Gillen D, Rubenstein A, Worcester E, Brecher ME, Asplin J, Coe F. Clinical decision support improves physician guideline adherence for laboratory monitoring of chronic kidney disease: a matched cohort study. BMC Nephrol. 2015 Oct 15;16:163. PMID: 26471846. doi: 10.1186/s12882-015-0159-5.

11. Garcia D, Moro CM, Cicogna PE, Carvalho DR. Method to integrate clinical guidelines into the electronic health record (EHR) by applying the archetypes approach. Stud Health Technol Inform. 2013;192:871-5. PMID: 23920682.

12. Gill JM, Chen YX, Grimes A, Diamond JJ, Lieberman MI, Klinkman MS. Electronic clinical decision support for management of depression in primary care: a prospective cohort study. Prim Care Companion CNS Disord. 2012;14(1). PMID: 22690364. doi: 10.4088/PCC.11m01191.

13. Gill JM, Chen YX, Grimes A, Klinkman MS. Using electronic health record-based tools to screen for bipolar disorder in primary care patients with depression. J Am Board Fam Med. 2012 May-Jun;25(3):283-90. PMID: 22570391. doi: 10.3122/jabfm.2012.03.110217.

14. Gill J, Kucharski K, Turk B, Pan C, Wei W. Using electronic clinical decision support in patient-centered medical homes to improve management of diabetes in primary care: The DECIDE study. J Ambul Care Manage. 2019 Apr/Jun;42(2):105-15. PMID: 30768429. doi: 10.1097/jac.0000000000000267.

15. Gudmundsson HT, Hansen KE, Halldorsson BV, Ludviksson BR, Gudbjornsson B. Clinical decision support system for the management of osteoporosis compared to NOGG guidelines and an osteology specialist: a validation pilot study. BMC Med Inform Decis Mak. 2019 Feb 1;19(1):27. PMID: 30709348. doi: 10.1186/s12911-019-0749-4.

16. Guenter D, Abouzahra M, Schabort I, Radhakrishnan A, Nair K, Orr S, Langevin J, Taenzer P, Moulin DE. Design process and utilization of a novel clinical decision support system for neuropathic pain in primary care: Mixed methods observational study. JMIR Med Inform. 2019 Sep 30;7(3):e14141. PMID: 31573946. doi: 10.2196/14141.

17. Gunathilake W, Gunawardena S, Fernando R, Thomson G, Fernando D. The impact of a decision support tool linked to an electronic medical record on glycemic control in people with type 2 diabetes. J Diabetes Sci Technol. 2013 May 1;7(3):653-9. PMID: 23759398. doi: 10.1177/193229681300700310.

18. Halldorsson BV, Bjornsson AH, Gudmundsson HT, Birgisson EO, Ludviksson BR, Gudbjornsson B. A clinical decision support system for the diagnosis, fracture risks and treatment of osteoporosis. Comput Math Methods Med. 2015;2015:189769. PMID: 25815042. doi: 10.1155/2015/189769.

19. Hendriks JML, Tieleman RG, Vrijhoef HJM, Wijtvliet P, Gallagher C, Prins MH, Sanders P, Crijns H. Integrated specialized atrial fibrillation clinics reduce all-cause mortality: post hoc analysis of a randomized clinical trial. Europace. 2019 Dec 1;21(12):1785-92. PMID: 31390464. doi: 10.1093/europace/euz209.

20. Heselmans A, Delvaux N, Laenen A, Van de Velde S, Ramaekers D, Kunnamo I, Aertgeerts B. Computerized clinical decision support system for diabetes in primary care does not improve quality of care: a cluster-randomized controlled trial. Implement Sci. 2020 Jan 7;15(1):5. PMID: 31910877. doi: 10.1186/s13012-019-0955-6.

21. Huang ES, Nathan AG, Cooper JM, Lee SM, Shin N, John PM, Dale W, Col NF, Meltzer DO, Chin MH. Impact and feasibility of personalized decision support for older patients with diabetes: A pilot randomized trial. Med Decis Making. 2017 Jul;37(5):611-7. PMID: 27311651. doi: 10.1177/0272989x16654142.

22. Karlsson LO, Nilsson S, Bång M, Nilsson L, Charitakis E, Janzon M. A clinical decision support tool for improving adherence to guidelines on anticoagulant therapy in patients with atrial fibrillation at risk of stroke: A cluster-randomized trial in a Swedish primary care setting (the CDS-AF study). PLoS Med. 2018 Mar;15(3):e1002528. PMID: 29534063. doi: 10.1371/journal.pmed.1002528.

23. Kropf M, Modre-Osprian R, Hayn D, Fruhwald F, Schreier G. Telemonitoring in heart failure patients with clinical decision support to optimize medication doses based on guidelines. Annu Int Conf IEEE Eng Med Biol Soc. 2014;2014:3168-71. PMID: 25570663. doi: 10.1109/embc.2014.6944295.

24. Litvin CB, Hyer JM, Ornstein SM. Use of clinical decision support to improve primary care identification and management of chronic kidney disease (CKD). J Am Board Fam Med. 2016 Sep-Oct;29(5):604-12. PMID: 27613793. doi: 10.3122/jabfm.2016.05.160020.

25. Lobach DF, Johns EB, Halpenny B, Saunders TA, Brzozowski J, Del Fiol G, Berry DL, Braun IM, Finn K, Wolfe J, Abrahm JL, Cooley ME. Increasing complexity in rule-based clinical decision support: The symptom assessment and management intervention. JMIR Med Inform. 2016 Nov 8;4(4):e36. PMID: 27826132. doi: 10.2196/medinform.5728.

26. Luo Y, Zhu Y, Chen J, Gao X, Yang W, Zou X, Zhou X, Ji L. A decision-support software to improve the standard care in chinese type 2 diabetes. J Diabetes Res. 2019;2019:5491743. PMID: 31828162. doi: 10.1155/2019/5491743.

27. Marcolino MS, Oliveira JAQ, Cimini CCR, Maia JX, Pinto V, Sá TQV, Amancio K, Coelho L, Ribeiro LB, Cardoso CS, Ribeiro AL. Development and implementation of a decision support system to improve control of hypertension and diabetes in a resource-constrained area in Brazil: Mixed methods study. J Med Internet Res. 2021 Jan 11;23(1):e18872. PMID: 33427686. doi: 10.2196/18872.

28. McKie PM, Kor DJ, Cook DA, Kessler ME, Carter RE, Wilson PM, Pencille LJ, Hickey BC, Chaudhry R. Computerized advisory decision support for cardiovascular diseases in primary care: A cluster randomized trial. Am J Med. 2020 Jun;133(6):750-6.e2. PMID: 31862329. doi: 10.1016/j.amjmed.2019.10.039.

29. Morganroth M, Pape G, Rozenfeld Y, Heffner JE. Multidisciplinary COPD disease management program: impact on clinical outcomes. Postgrad Med. 2016;128(2):239-49. PMID: 26641555. doi: 10.1080/00325481.2016.1129259.

30. O'Connor PJ, Sperl-Hillen JM, Rush WA, Johnson PE, Amundson GH, Asche SE, Ekstrom HL, Gilmer TP. Impact of electronic health record clinical decision support on diabetes care: a randomized trial. Ann Fam Med. 2011 Jan-Feb;9(1):12-21. PMID: 21242556. doi: 10.1370/afm.1196.

31. Orchard J, Li J, Freedman B, Webster R, Salkeld G, Hespe C, Gallagher R, Patel A, Kamel B, Neubeck L, Lowres N. Atrial fibrillation screen, management, and guideline-recommended therapy in the rural primary care setting: A cross-sectional study and cost-effectiveness analysis of eHealth tools to support all stages of screening. J Am Heart Assoc. 2020 Sep 15;9(18):e017080. PMID: 32865129. doi: 10.1161/jaha.120.017080.

32. Popescu C, Golden G, Benrimoh D, Tanguay-Sela M, Slowey D, Lundrigan E, Williams J, Desormeau B, Kardani D, Perez T, Rollins C, Israel S, Perlman K, Armstrong C, Baxter J, Whitmore K, Fradette MJ, Felcarek-Hope K, Soufi G, Fratila R, Mehltretter J, Looper K, Steiner W, Rej S, Karp JF, Heller K, Parikh SV, McGuire-Snieckus R, Ferrari M, Margolese H, Turecki G. Evaluating the clinical feasibility of an artificial intelligence-powered, web-based clinical decision support system for the treatment of depression in adults: Longitudinal feasibility study. JMIR Form Res. 2021 Oct 25;5(10):e31862. PMID: 34694234. doi: 10.2196/31862.

33. Prabhakaran D, Jha D, Prieto-Merino D, Roy A, Singh K, Ajay VS, Jindal D, Gupta P, Kondal D, Goenka S, Jacob P, Singh R, Kumar BGP, Perel P, Tandon N, Patel V. Effectiveness of an mHealth-based electronic decision support system for integrated management of chronic conditions in primary care: The mWellcare cluster-randomized controlled trial. Circulation. 2019 Jan 15;139(3):380-91. PMID: 30586732. doi: 10.1161/circulationaha.118.038192.

34. Regan ME. Implementing an evidence-based clinical decision support tool to improve the detection, evaluation, and referral patterns of adult chronic kidney disease patients in primary care. J Am Assoc Nurse Pract. 2017 Dec;29(12):741-53. PMID: 28875593. doi: 10.1002/2327-6924.12505.

35. O'Reilly D, Holbrook A, Blackhouse G, Troyan S, Goeree R. Cost-effectiveness of a shared computerized decision support system for diabetes linked to electronic medical records. J Am Med Inform Assoc. 2012 May-Jun;19(3):341-5. PMID: 22052900. doi: 10.1136/amiajnl-2011-000371.

36. Reynolds EL, Burke JF, Banerjee M, Callaghan BC. Randomized controlled trial of a clinical decision support system for painful polyneuropathy. Muscle Nerve. 2020 May;61(5):640-4. PMID: 31811650. doi: 10.1002/mus.26774.

37. Rieckert A, Reeves D, Altiner A, Drewelow E, Esmail A, Flamm M, Hann M, Johansson T, Klaassen-Mielke R, Kunnamo I, Löffler C, Piccoliori G, Sommerauer C, Trampisch US, Vögele A, Woodham A, Sönnichsen A. Use of an electronic decision support tool to reduce polypharmacy in elderly people with chronic diseases: cluster randomised controlled trial. BMJ. 2020 Jun 18;369:m1822. PMID: 32554566. doi: 10.1136/bmj.m1822.

38. Robinson DG, Schooler NR, Correll CU, John M, Kurian BT, Marcy P, Miller AL, Pipes R, Trivedi MH, Kane JM. Psychopharmacological treatment in the RAISE-ETP Study: Outcomes of a manual and computer decision support system based intervention. Am J Psychiatry. 2018 Feb 1;175(2):169-79. PMID: 28945118. doi: 10.1176/appi.ajp.2017.16080919.

39. Rodbard HW, Schnell O, Unger J, Rees C, Amstutz L, Parkin CG, Jelsovsky Z, Wegmann N, Axel-Schweitzer M, Wagner RS. Use of an automated decision support tool optimizes clinicians' ability to interpret and appropriately respond to structured self-monitoring of blood glucose data. Diabetes Care. 2012 Apr;35(4):693-8. PMID: 22344611. doi: 10.2337/dc11-1351.

40. Rossom RC, Crain AL, O'Connor PJ, Waring SC, Hooker SA, Ohnsorg K, Taran A, Kopski KM, Sperl-Hillen JM. Effect of clinical decision support on cardiovascular risk among adults with bipolar disorder, schizoaffective disorder, or schizophrenia: A cluster randomized clinical trial. JAMA Netw Open. 2022 Mar 1;5(3):e220202. PMID: 35254433. doi: 10.1001/jamanetworkopen.2022.0202.

41. Scheitel MR, Kessler ME, Shellum JL, Peters SG, Milliner DS, Liu H, Komandur Elayavilli R, Poterack KA, Miksch TA, Boysen J, Hankey RA, Chaudhry R. Effect of a novel clinical decision support tool on the efficiency and accuracy of treatment recommendations for cholesterol management. Appl Clin Inform. 2017 Feb 8;8(1):124-36. PMID: 28174820. doi: 10.4338/aci-2016-07-ra-0114.

42. Sim LL, Ban KH, Tan TW, Sethi SK, Loh TP. Development of a clinical decision support system for diabetes care: A pilot study. PLoS One. 2017;12(2):e0173021. PMID: 28235017. doi: 10.1371/journal.pone.0173021.

43. Sperl-Hillen JM, Crain AL, Margolis KL, Ekstrom HL, Appana D, Amundson G, Sharma R, Desai JR, O'Connor PJ. Clinical decision support directed to primary care patients and providers reduces cardiovascular risk: a randomized trial. J Am Med Inform Assoc. 2018 Sep 1;25(9):1137-46. PMID: 29982627. doi: 10.1093/jamia/ocy085.

44. Vromen T, Peek NB, Abu-Hanna A, Kornaat M, Kemps HM. A computerized decision support system did not improve personalization of exercise-based cardiac rehabilitation according to latest recommendations. Eur J Prev Cardiol. 2021 May 14;28(5):572-80. PMID: 33624044. doi: 10.1093/eurjpc/zwaa066.

45. Wagholikar KB, MacLaughlin KL, Henry MR, Greenes RA, Hankey RA, Liu H, Chaudhry R. Clinical decision support with automated text processing for cervical cancer screening. J Am Med Inform Assoc. 2012 Sep-Oct;19(5):833-9. PMID: 22542812. doi: 10.1136/amiajnl-2012-000820.

46. Wagholikar KB, MacLaughlin KL, Kastner TM, Casey PM, Henry M, Greenes RA, Liu H, Chaudhry R. Formative evaluation of the accuracy of a clinical decision support system for cervical cancer screening. J Am Med Inform Assoc. 2013 Jul-Aug;20(4):749-57. PMID: 23564631. doi: 10.1136/amiajnl-2013-001613.

47. Wijtvliet E, Tieleman RG, van Gelder IC, Pluymaekers N, Rienstra M, Folkeringa RJ, Bronzwaer P, Elvan A, Elders J, Tukkie R, Luermans J, Van Asselt A, Van Kuijk SMJ, Tijssen JG, Crijns H. Nurse-led vs. usual-care for atrial fibrillation. Eur Heart J. 2020 Feb 1;41(5):634-41. PMID: 31544925. doi: 10.1093/eurheartj/ehz666.

48. Xu F, Sepúlveda MJ, Jiang Z, Wang H, Li J, Yin Y, Liu Z, Roebuck MC, Shortliffe EH, Yan M, Song Y, Geng C, Tang J, Rhee K. Artificial intelligence treatment decision support for complex breast cancer among oncologists with varying expertise. JCO Clin Cancer Inform. 2019 Aug;3:1-15. PMID: 31419181. doi: 10.1200/cci.18.00159.

49. Xu F, Sepúlveda MJ, Jiang Z, Wang H, Li J, Liu Z, Yin Y, Roebuck MC, Shortliffe EH, Yan M, Song Y, Geng C, Tang J, Purcell Jackson G, Preininger AM, Rhee K. Effect of an artificial intelligence clinical decision support system on treatment decisions for complex breast cancer. JCO Clin Cancer Inform. 2020 Sep;4:824-38. PMID: 32970484. doi: 10.1200/cci.20.00018.
