# Supplementary material for: Benefits of Clinical Decision Support Systems for the Management of Noncommunicable Chronic Diseases: Targeted Literature Review
Source: Interact J Med Res. 2024 Nov 27;13:e58036. doi: 10.2196/58036 (PMC11635333; doi:10.2196/58036)
Supplement: Multimedia Appendix 6 [file ijmr_v13i1e58036_app6.docx]

**Multimedia Appendix 6: Features of CDSSs for All Successful Studies**

**Figure S1. Frequency of features for all successful studies**

**Figure S2. Frequency of features for successful stand-alone studies**

**Figure S3. Frequency of features for successful integrated studies**

Abbreviation: rec, recommendation
